# Identification of soluble biomarkers that associate with distinct manifestations of long COVID

**DOI:** 10.1038/s41590-025-02135-5

**Published:** 2025-04-30

**Authors:** Yu Gao, Curtis Cai, Sarah Adamo, Elsa Biteus, Habiba Kamal, Lena Dager, Kelly L. Miners, Sian Llewellyn-Lacey, Kristin Ladell, Pragati S. Amratia, Kirsten Bentley, Simon Kollnberger, Jinghua Wu, Mily Akhirunnesa, Samantha A. Jones, Per Julin, Christer Lidman, Richard J. Stanton, Paul A. Goepfert, Michael J. Peluso, Steven G. Deeks, Helen E. Davies, Soo Aleman, Marcus Buggert, David A. Price

**Affiliations:** 1 Center for Infectious Medicine, Department of Medicine Huddinge, Karolinska Institutet, Stockholm, Sweden; 2 Laboratory of Translational Immuno-Oncology, Department of Biomedicine, University and University Hospital Basel, Basel, Switzerland; 3 Division of Infectious Diseases and Dermatology, Department of Medicine Huddinge, Karolinska Institutet, Stockholm, Sweden; 4 Center for Clinical Research Sörmland, Uppsala University, Uppsala, Sweden; 5 Department of Infectious Diseases, Karolinska University Hospital, Stockholm, Sweden; 6 Division of Infection and Immunity, Cardiff University School of Medicine, University Hospital of Wales, Cardiff, UK; 7 Department of Respiratory Medicine, University Hospital Llandough, Penarth, UK; 8 Post-COVID Policlinic, Karolinska University Hospital, Stockholm, Sweden; 9 Department of Neurobiology, Care Sciences and Society, Karolinska Institutet, Stockholm, Sweden; 10 Division of Infectious Diseases, Department of Medicine, University of Alabama at Birmingham, Birmingham, AL USA; 11 Department of Medicine, University of California, San Francisco, San Francisco, CA USA; 12 Systems Immunity Research Institute, Cardiff University School of Medicine, University Hospital of Wales, Cardiff, UK

**Keywords:** Viral infection, Viral infection

## Abstract

Long coronavirus disease (COVID) is a heterogeneous clinical condition of uncertain etiology triggered by infection with severe acute respiratory syndrome coronavirus 2 (SARS-CoV-2). Here we used ultrasensitive approaches to profile the immune system and the plasma proteome in healthy convalescent individuals and individuals with long COVID, spanning geographically independent cohorts from Sweden and the United Kingdom. Symptomatic disease was not consistently associated with quantitative differences in immune cell lineage composition or antiviral T cell immunity. Healthy convalescent individuals nonetheless exhibited higher titers of neutralizing antibodies against SARS-CoV-2 than individuals with long COVID, and extensive phenotypic analyses revealed a subtle increase in the expression of some co-inhibitory receptors, most notably PD-1 and TIM-3, among SARS-CoV-2 nonspike-specific CD8^+^ T cells in individuals with long COVID. We further identified a shared plasma biomarker signature of disease linking breathlessness with apoptotic inflammatory networks centered on various proteins, including CCL3, CD40, IKBKG, IL-18 and IRAK1, and dysregulated pathways associated with cell cycle progression, lung injury and platelet activation, which could potentially inform the diagnosis and treatment of long COVID.

## Abstract (web summary)

Gao and colleagues explore soluble biomarkers, immune cell subsets, viral antibody responses and plasma proteomics to better define and understand long COVID in two independent cohorts.

## Main

Severe acute respiratory syndrome coronavirus 2 (SARS-CoV-2) has left a pernicious legacy of global ill health, commonly known as long coronavirus disease (COVID)^1^. The etiology of this heterogeneous condition remains obscure, but common symptoms include breathlessness, cognitive impairment, often described as ‘brain fog’, fatigue and pain, alongside a host of other clinical manifestations indicating the involvement of different organ systems in the body^2,3^. Several hypotheses have been proposed to account for such diverse and persistent symptomatology, including immune dysregulation, ongoing inflammation and tissue damage, and viral persistence^3–9^. The reactivation of latent herpesviruses may also contribute to the pathogenesis of long COVID^3,10,11^.

A handful of ‘omics approaches have been used to probe the molecular intricacies of long COVID. For example, affinity proteomics studies have identified distinct inflammatory phenotypes and enrichment of the NF-κB and type 2 interferon (IFN) signaling pathways as correlates of disease, highlighting associations with various soluble biomarkers, such as IFNγ, interleukin-1 (IL-1), IL-6 and tumor necrosis factor (TNF), which are typically upregulated in acute COVID-19 (refs. ^12–14^). Similar findings have been described using conventional approaches to cytokine quantification^15^. A longitudinal multiomics study further reported that various autoantibodies, altered cytomegalovirus (CMV)-specific and SARS-CoV-2-specific CD8^+^ T cell dynamics, and Epstein–Barr virus (EBV) and SARS-CoV-2 viremia were associated with the emergence of particular subtypes of long COVID^10^. More recently, another multiomics study found that elevated herpesvirus-specific antibody titers, immune cell perturbations, and decreased cortisol levels were distinguishing features of persistent illness after infection with SARS-CoV-2 (ref. ^16^). By contrast, hypothesis-driven approaches and low-resolution proteomics have identified dysregulation of the complement system, which is known to drive inflammation, as a consistent feature of long COVID^17,18^. These observations suggest that multiple factors could be associated with the development of discrete symptom complexes and patterns of disease onset within the clinically diverse spectrum of long COVID.

In this study, we used a variety of multidimensional approaches and integrative data analysis pipelines to profile the immune system and the plasma proteome in healthy convalescent individuals with a molecularly confirmed history of infection with SARS-CoV-2 and individuals with long COVID, spanning geographically independent cohorts from Sweden and the United Kingdom. We found higher titers of SARS-CoV-2 spike-specific neutralizing antibodies in healthy convalescent individuals than in individuals with long COVID. By contrast, minimal intergroup differences were apparent in immune cell lineage composition and virus-specific CD4^+^ and CD8^+^ T cell immunity, although some co-inhibitory receptors, especially PD-1 and TIM-3, were relatively overexpressed among SARS-CoV-2 nonspike-specific CD8^+^ T cells in individuals with long COVID. We also detected a unique array of soluble biomarkers in the plasma proteome that correlated directly with the clinical manifestation of breathlessness in individuals with long COVID. Network and pathway analyses linked these biomarker signatures with apoptotic processes and inflammation, highlighting key roles for signaling cascades involving ceramide, FAS, NF-κB and TNF. Moreover, core network components, including CCL3, CD40 and IL-18, were identified as potential contributors to persistent inflammation in individuals with long COVID. These results provide a mechanistic framework to unravel the complex etiology and pathogenesis of ongoing symptomatic disease triggered by infection with SARS-CoV-2.

## Results

### Clinical characterization

**Fig. 1:**
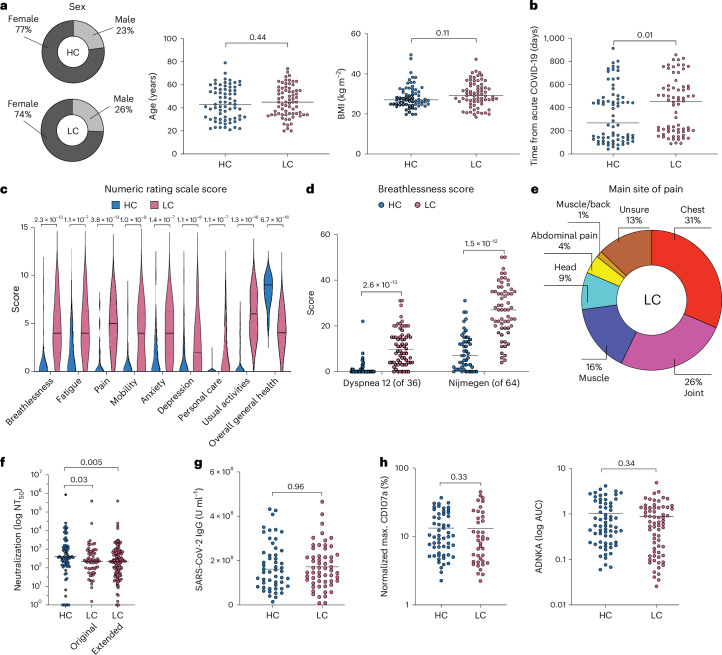
Cohort overview and humoral immunity in healthy convalescent individuals and individuals with long COVID recruited from the United Kingdom. **a**, Ring charts and scatter dot plots showing sex, age and BMI for healthy convalescent individuals (HC, *n* = 70) and individuals with long COVID (LC, *n* = 70). **b**, Scatter dot plots showing the corresponding time to sampling from the initial diagnosis of acute COVID-19. **c**, Violin plots showing the corresponding distribution of clinical symptom numeric rating scale scores. **d**, Scatter dot plots showing breathlessness scores as assessed using the Dyspnea-12 and Nijmegen questionnaires (HC, *n* = 49 and *n* = 49, respectively; LC, *n* = 66 and *n* = 62, respectively). **e**, Ring chart highlighting the anatomical distribution of pain experienced by individuals with long COVID. **f**, Scatter dot plot showing SARS-CoV-2-specific neutralization activity quantified as the highest plasma dilution that achieved a 50% reduction in plaque formation (NT_50_; HC, *n* = 70; LC, original *n* = 70 and extended *n* = 146). **g**, Scatter dot plot showing total SARS-CoV-2 spike-specific immunoglobulin titers (HC, *n* = 52; LC, *n* = 57). **h**, Scatter dot plots showing maximum CD107a mobilization (left) quantified as percentage values relative to the corresponding positive controls and normalized ADNKA (right) quantified as a function of degranulation (CD107a^+^) among viable NK cells (Aqua^−^CD3^−^CD56^+^) with potent cytotoxic activity (CD57^+^; HC, *n* = 55 and *n* = 66, respectively; LC, *n* = 40 and *n* = 66, respectively); AUC, area under the curve. Horizontal bars represent median values (**a**–**d** and **f**–**h**). Significance was evaluated using a two-tailed Mann–Whitney *U*-test (**a**–**d** and **f**–**h**).

The primary cohort included healthy convalescent individuals (controls; *n* = 70) and individuals with long COVID (cases; *n* = 70) recruited from University Hospital Llandough (Table 1 and Supplementary Table 1). All participants had a clearly defined episode of symptomatically mild acute COVID-19 confirmed via direct molecular evidence of infection with SARS-CoV-2. Intergroup comparisons revealed largely equivalent distributions for age (cases, median = 45 years; controls, median = 43 years), body mass index (BMI; cases, median = 29.8 kg m^–2^; controls, median = 28.5 kg m^–2^), race (cases, White = 88.6%; controls, White = 82.9%), sex (cases, female = 74.3%; controls, female = 77.2%), time since initial reported infection (cases, median = 416 days; controls, median = 268 days), and vaccination against SARS-CoV-2 (cases, median number of vaccinations = 3; controls, median number of vaccinations = 3; Fig. 1a,b and Table 1). All baseline medical evaluations were normal in individuals with long COVID. Symptom scores are depicted in Fig. 1c. Breathlessness was further assessed using the Dyspnea-12 questionnaire, scored out of 36, and the Nijmegen questionnaire, scored out of 64, which provide a metric for hyperventilation (Fig. 1d). Pain was most commonly localized to the chest (31%), joints (26%) and muscles (16%) in individuals with long COVID (Fig. 1e). The secondary cohort included healthy convalescent individuals (controls; *n* = 30) and individuals with long COVID (cases; *n* = 95) recruited from Karolinska University Hospital (Table 2).

**Table 1:** Key features of the primary cohort recruited from the United Kingdom

Characteristic	Controls (*n* = 70)	Cases (*n* = 70)
Age (years), median (range)	43 (21–79)	45 (20–74)
Female (%)	54 (77.1)	52 (74.3)
Race (%)		
White	58 (82.9)	62 (88.6)
Black	0 (0.0)	1 (1.4)
Asian	9 (12.9)	4 (5.7)
Mixed	2 (2.9)	2 (2.9)
Other	1 (1.4)	1 (1.4)
BMI > 30 kg m^–2^ (%)	21/66 (31.8)	32/69 (46.3)
Coexisting conditions (%)		
Yes	13 (18.6)	35 (50.0)
No	57 (81.4)	35 (50.0)
Employment status (%)		
Pre-COVID-19		
Employed	45/49 (91.8)	59/66 (89.4)
Employed, altered duties	0/49 (0.0)	0/66 (0.0)
Employed, sick leave	0/49 (0.0)	0/66 (0.0)
Unemployed	0/49 (0.0)	1/66 (1.5)
Retired	1/49 (2.0)	4/66 (6.1)
Student	3/49 (6.1)	2/66 (3.0)
Post-COVID-19		
Employed	46/49 (93.9)	35/66 (53.0)
Employed, altered duties	0/49 (0.0)	11/66 (16.7)
Employed, sick leave	0/49 (0.0)	11/66 (16.7)
Unemployed	0/49 (0.0)	2/66 (3.0)
Retired	1/49 (2.0)	5/66 (7.6)
Student	2/49 (4.1)	2/66 (3.0)
Date of infection with SARS-CoV-2 (%)		
March 2020 to August 2020	11 (15.7)	14 (20.0)
September 2020 to June 2021	23 (32.9)	25 (35.7)
July 2021 to October 2022	36 (51.4)	31 (44.3)
COVID-19 vaccination status, median (IQR)		
Number of vaccinations before infection	1.5 (0–3)	0 (0–2)
Total number of vaccinations	3 (3–3)	3 (3–3)
Symptoms, median (IQR)^a^		
Breathlessness	0 (0–0)	3 (2–6)
Fatigue	0 (0–2)	6 (4–8)
Musculoskeletal	0 (0–0)	3.5 (0–6)
Neuropsychiatric	0 (0–0)	4 (1–6)
Pain	0 (0–0)	4 (2–5)
Ability to maintain self-care, median (IQR)	0 (0–0)	0 (0–3)
Ability to maintain daily tasks, median (IQR)	0 (0–0)	5 (3–8)
Overall general health, median (IQR)^b^	0 (−1 to 0)	−4 (−2 to −6)

IQR, interquartile range.

^a^Difference in numeric rating scale score (0 = no symptom, 10 = worst possible symptom) before versus after COVID-19.

^b^Difference in numeric rating scale score (0 = worst possible, 10 = best possible) before versus after COVID-19.

**Table 2:** Key features of individuals with long COVID recruited from Sweden

Characteristic	Borg CR10 ≤ 2 (*n* = 60)	Borg CR10 > 2 (*n* = 35)
Age (years), median (range)	46 (21–64)	50 (23–66)
Female (%)	88.7%	75.6%
BMI > 30 kg m^–2^ (%)	15/41 (22.9)	19/45 (27.2)

### Neutralizing antibody titers are suboptimal in long COVID

To evaluate the humoral immune system, we measured total SARS-CoV-2 spike-specific immunoglobulin titers, virus neutralization activity, and antibody-dependent natural killer (NK) cell activation (ADNKA) in plasma samples obtained from donors in the United Kingdom. Healthy convalescent individuals exhibited substantially better neutralization activity in standard plaque reduction assays than individuals with long COVID (Fig. 1f), despite equivalent overall titers of antibodies targeting the spike protein of SARS-CoV-2 (Fig. 1g). This finding was confirmed across a larger number of donors from the same cohort^17^, achieving even greater significance (Fig. 1f). By contrast, no such intergroup differences were apparent for ADNKA measured as a cumulative metric against all expressed viral target proteins using healthy donor cell preparations with a surrogate marker of potential cytotoxicity (Fig. 1h), namely CD57 (ref. ^19^).

Collectively, these findings identify a qualitative deficit in the humoral immune response against SARS-CoV-2, specifically impacting neutralization activity in individuals with long COVID.

### Immune cell perturbations are limited in long COVID

**Fig. 2:**
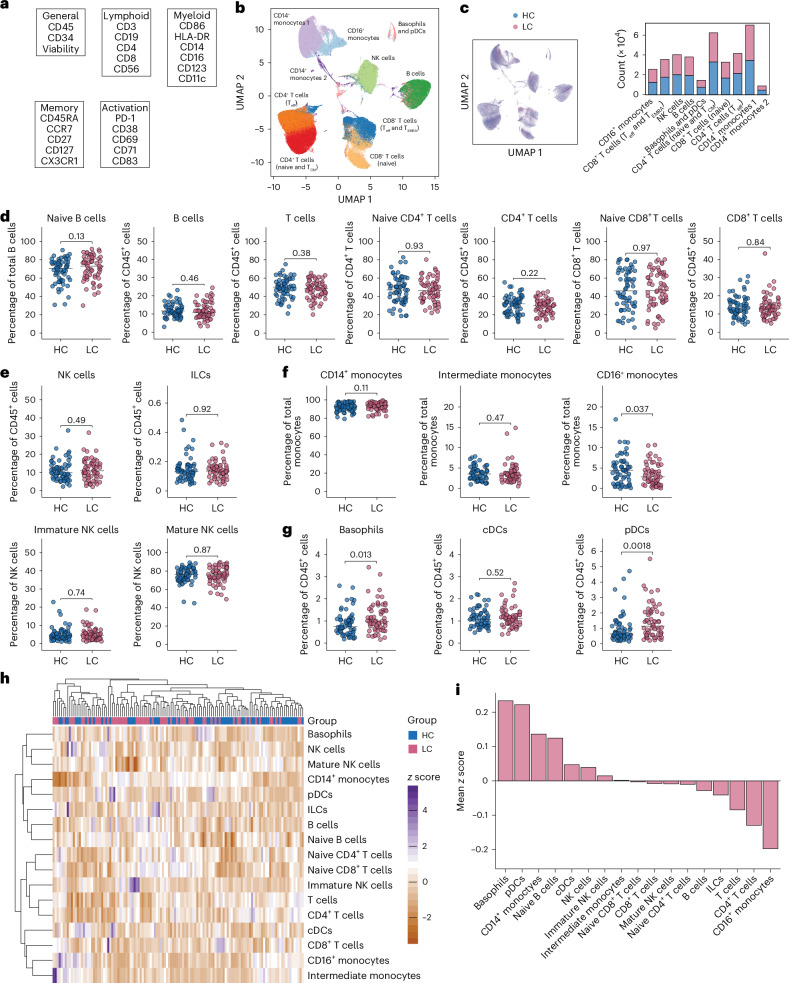
Immune cell lineages in healthy convalescent individuals and individuals with long COVID recruited from the United Kingdom. **a**, List of surface markers used to characterize immune cell lineages in the periphery. **b**, Uniform manifold approximation and projection (UMAP) representation of immune cell lineages identified via dimensionality reduction of marker expression values; T_eff_, effector T cells; T_EMRA_, terminally differentiated effector memory T cells; T_CM_, central memory T cells. **c**, Distribution of cells by group of origin in UMAP space (left) or within UMAP clusters (right). **d**, Scatter dot plots showing the frequencies of naive and total B and T cells gated manually. **e**, Scatter dot plots showing the frequencies of innate lymphocytes gated manually; ILCs, innate lymphoid cells. **f**, Scatter dot plots showing the frequencies of monocytes gated manually. **g**, Scatter dot plots showing the frequencies of basophils and DCs gated manually; cDCs, conventional DCs. **h**, Heat map showing hierarchically clustered *z* scores derived from the frequencies of immune cell subsets gated manually. **i**, Bar plot showing mean *z* scores for each immune cell subset gated manually for individuals with long COVID (healthy convalescent individuals, *n* ≤ 70; individuals with long COVID, *n* ≤ 70; **b**–**i**). Horizontal bars represent median values (**d**–**g**). Significance was evaluated using a two-tailed Mann–Whitney *U*-test (**d**–**g**).

**Extended Data Fig. 1:**
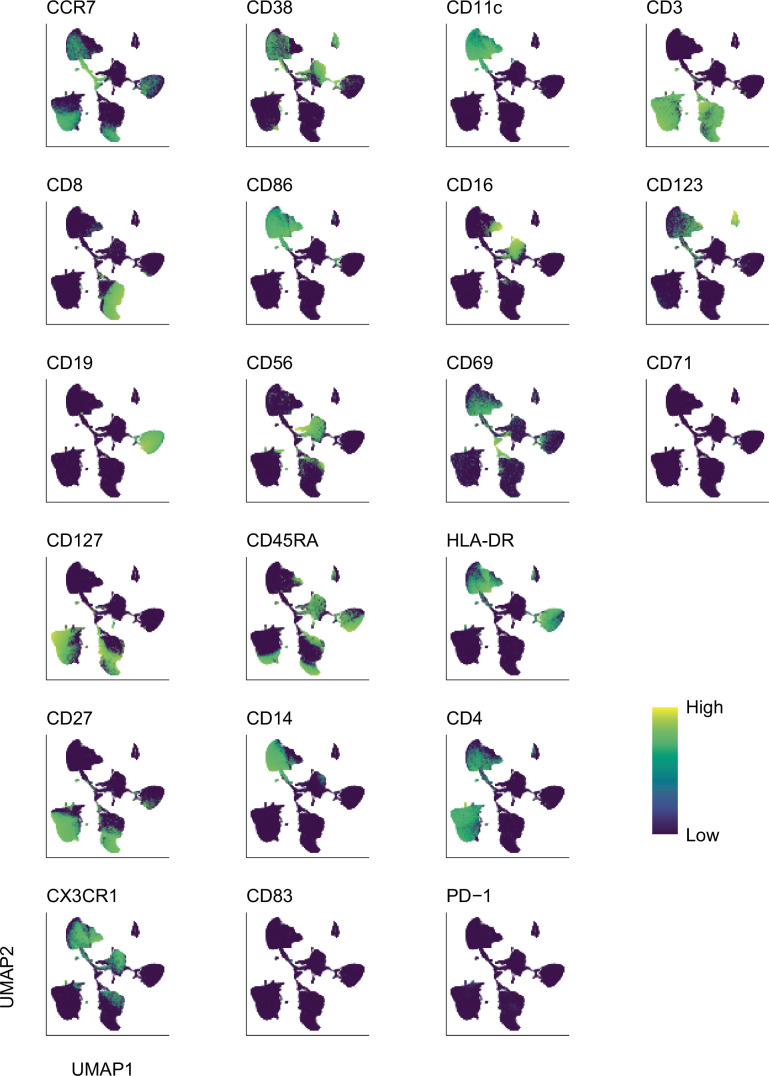
Expression of immune cell lineage markers measured via flow cytometry. UMAP representation of individual immune cell lineage markers among peripheral blood mononuclear cells after dimensionality reduction.

**Extended Data Fig. 2:**
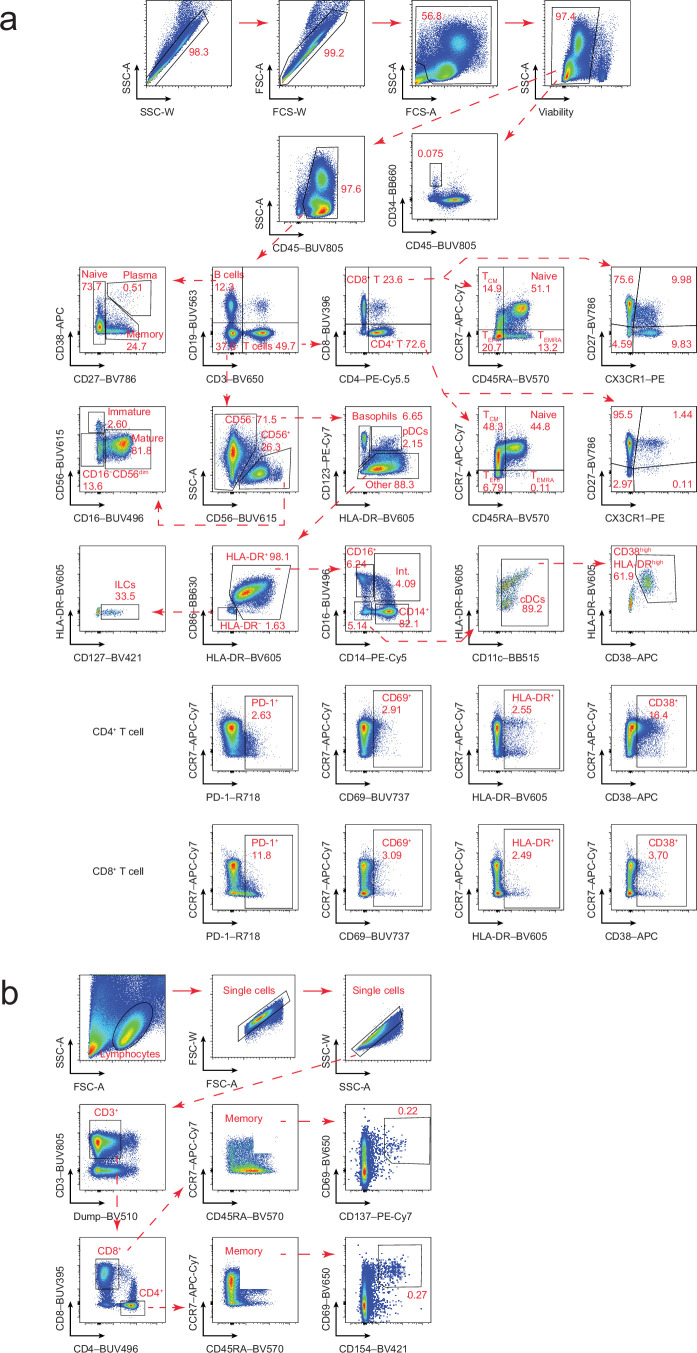
Flow cytometric gating strategies for the identification of immune cell lineages and activation-induced marker upregulation. (**a**) Flow cytometric gating strategy for immune cell lineage characterization. Numbers indicate percentages in the drawn gates. (**b**) Flow cytometric gating strategy for the identification of antigen-specific CD4^+^ and CD8^+^ T cells via upregulation of the activation-induced markers CD69 and CD154 or CD69 and CD137, respectively. Numbers indicate percentages in the drawn gates.

To evaluate the cellular immune system, we first conducted a multidimensional flow cytometric analysis of the major lineages typically present among peripheral blood mononuclear cells (PBMCs), focusing initially on donors recruited from the United Kingdom (Fig. 2a). Using dimensionality reduction and Gaussian mixture models, we identified clusters that corresponded to the major lineages of monocytes, B cells, NK cells and T cells (Fig. 2b and Extended Data Fig. 1), but these analyses were unable to differentiate between some other immune cell subsets, such as basophils and plasmacytoid dendritic cells (pDCs; Fig. 2b), and were also unable to differentiate between healthy convalescent individuals and individuals with long COVID (Fig. 2c). We therefore interrogated the data conventionally using a manual flow cytometric gating strategy (Extended Data Fig. 2a).

**Extended Data Fig. 3:**
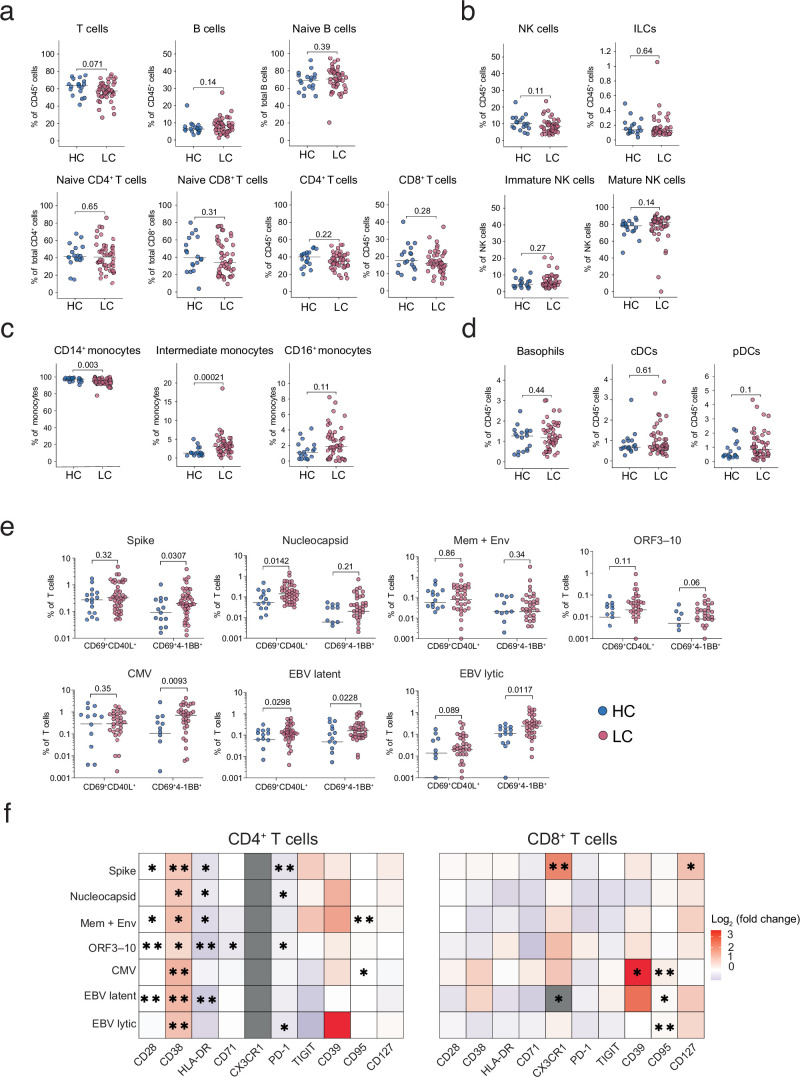
Immune cell lineages and T cell immunity in healthy convalescent individuals and patients with long COVID recruited from Sweden. (**a**) Scatter dot plots showing the frequencies of naive and total B and T cells gated manually. (**b**) Scatter dot plots showing the frequencies of innate lymphocytes gated manually. (**c**) Scatter dot plots showing the frequencies of monocytes gated manually. (**d**) Scatter dot plots showing the frequencies of basophils and dendritic cells gated manually. (**e**) Scatter dot plots showing the frequencies of functional CD4^+^ and CD8^+^ T cells targeting defined proteins from SARS-CoV-2, CMV, or EBV. (**f**) Heatmap summarizing the phenotypic attributes of functional CD4^+^ and CD8^+^ T cells targeting defined proteins from SARS-CoV-2, CMV, or EBV. Data are shown for each marker as the log_2_-transformed fold change in percent positive for each population among patients with long COVID (LC) versus healthy convalescent individuals (HC). *P < 0.05, **P < 0.01. HC, *n* ≤ 20; LC, *n* ≤ 56 (**a, b, c, d, e** and **f**). Horizontal bars represent median values (**a**, **b**, **c**, **d** and **e**). Significance was evaluated using a two-tailed Mann–Whitney U test (**a**, **b**, **c**, **d**, **e** and **f**).

In the adaptive lymphocyte compartment, similar proportions of naive B cells, total B cells, naive T cells, total T cells, naive CD4^+^ T cells, total CD4^+^ T cells, naive CD8^+^ T cells and total CD8^+^ T cells were identified in healthy convalescent individuals and individuals with long COVID (Fig. 2d), and in the innate lymphocyte compartment, similar proportions of immature NK cells (CD16^−^CD56^bright^), mature NK cells (CD16^+^CD56^dim^), total NK cells (including CD16^−^CD56^dim^) and total innate lymphoid cells (CD127^+^) were identified in healthy convalescent individuals and individuals with long COVID (Fig. 2e). A comparable pattern was observed for classical monocytes (CD14^+^), intermediate monocytes (CD14^+^CD16^+^) and conventional DCs (CD11c^+^CD123^−^) in the myeloid cell lineage, whereas the proportions of nonclassical monocytes (CD16^+^) were relatively increased in healthy convalescent individuals (Fig. 2f), and the proportions of basophils (CD123^+^HLA-DR^−^) and pDCs (CD123^+^HLA-DR^+^) were relatively increased in individuals with long COVID (Fig. 2g). Hierarchical clustering confirmed these differences within an otherwise rather uniform immune cell landscape (Fig. 2h,i). By contrast, no such perturbations were apparent in the secondary cohort of donors recruited from Sweden, although the proportions of classical monocytes were relatively decreased and the proportions of intermediate monocytes were relatively increased in individuals with long COVID (Extended Data Fig. 3a–d).

Collectively, these data indicate that immune cell perturbations are quantitatively subtle and, despite intercohort variability, generally confined to the myeloid compartment in individuals with long COVID.

### T cell immunity remains largely unaltered in long COVID

**Fig. 3:**
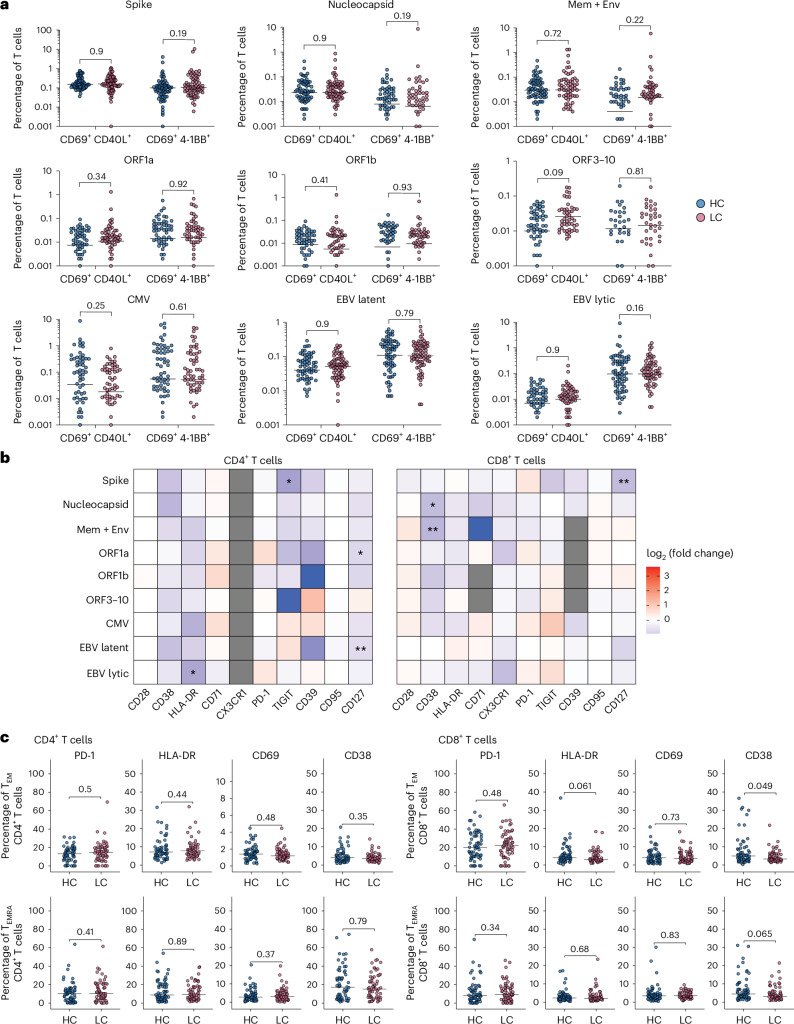
T cell immunity in healthy convalescent individuals and individuals with long COVID recruited from the United Kingdom. **a**, Scatter dot plots showing the frequencies of functional CD4^+^ and CD8^+^ T cells targeting defined proteins from SARS-CoV-2, CMV or EBV; Mem, membrane; Env, envelope. **b**, Heat map summarizing the phenotypic attributes of functional CD4^+^ and CD8^+^ T cells targeting defined proteins from SARS-CoV-2, CMV or EBV. Data are shown for each marker as the log_2_-transformed fold change in percent positive for each population among individuals with long COVID versus healthy convalescent individuals; *P < 0.05 and **P < 0.01. **c**, Scatter dot plots showing the frequencies of functional CD4^+^ and CD8^+^ effector memory T (T_EM_; top) cells and terminally differentiated effector memory T (T_EMRA_; bottom) cells expressing the indicated activation markers; healthy convalescent individuals, *n* ≤ 70; individuals with long COVID, *n* ≤ 70 (**a**–**c**). Horizontal bars represent median values (**a** and **c**). Significance was evaluated using a two-tailed Mann–Whitney *U*-test (**a**–**c**).

To extend these findings, we quantified CD4^+^ and CD8^+^ memory T cell responses against SARS-CoV-2 and the persistent herpesviruses CMV and EBV, exposure to which has been differentially linked with the development of long COVID^10,16,20,21^. We used activation-induced marker (AIM) assays for this purpose, enumerating functional antigen-specific CD4^+^ T cells by assessing the upregulation of CD69 and CD40L (CD154) and functional antigen-specific CD8^+^ T cells by assessing the upregulation of CD69 and 4-1BB (CD137) after peptide stimulation directly ex vivo^22,23^. PBMCs were stimulated with individual peptide pools spanning the major immunogenic proteins from SARS-CoV-2 (spike, nucleocapsid, combined membrane and envelope, ORF1a, ORF1b and ORF3–ORF10) and selected immunogenic proteins from CMV (IE-1, IE-2 and pp65) and EBV, the latter segregated according to lytic (BRLF1, BZLF1, BMLF1 and BARF1) and latent phases (EBNA1, EBNA2, EBNA3A, EBNA3B, EBNA3C and LMP2) of the viral life cycle (Extended Data Fig. 2b). The frequencies of antiviral CD4^+^ and CD8^+^ T cells were statistically indistinguishable across all of these specificities in healthy convalescent individuals and individuals with long COVID recruited from the United Kingdom (Fig. 3a). By contrast, the frequencies of CD4^+^ T cells targeting the SARS-CoV-2 nucleocapsid protein and the EBV latent proteins and the frequencies of CD8^+^ T cells targeting the SARS-CoV-2 spike protein, the CMV proteins and the EBV lytic proteins were higher in individuals with long COVID than in healthy convalescent individuals recruited from Sweden (Extended Data Fig. 3e).

In further experiments, we measured the expression of immunophenotypic markers related to activation, memory, effector function and exhaustion among CD4^+^ and CD8^+^ T cells targeting defined proteins from SARS-CoV-2, CMV or EBV. In the primary cohort, no significant intergroup differences in expression intensity were observed for CD28, CD39, CD71, CD95, CX3CR1 or PD-1, but some markers of activation (CD38 and HLA-DR), exhaustion (TIGIT) and stemness (CD127) were variably downregulated among some antiviral CD4^+^ and CD8^+^ T cell populations in the context of long COVID (Fig. 3b). More profound differences were apparent in the secondary cohort, potentially reflecting the limited number of healthy convalescent individuals relative to the number of individuals with long COVID (Extended Data Fig. 3f). Lineage analysis further revealed comparable expression of CD38, CD69, HLA-DR and PD-1 among global CD4^+^ and CD8^+^ effector memory T cells and terminally differentiated effector memory T cells in healthy convalescent individuals and individuals with long COVID recruited from the United Kingdom (Fig. 3c).

Collectively, these results demonstrate that experimental findings are not necessarily transferable across geographically distinct cohorts of individuals with long COVID, likely reflecting differences in clinical characterization and sample size. Our findings nonetheless align with the notion that circulating antiviral CD4^+^ and CD8^+^ T cell populations are largely equivalent in healthy convalescent individuals and individuals with long COVID^24^.

### SARS-CoV-2-specific CD8^+^ T cells are phenotypically variable

**Fig. 4:**
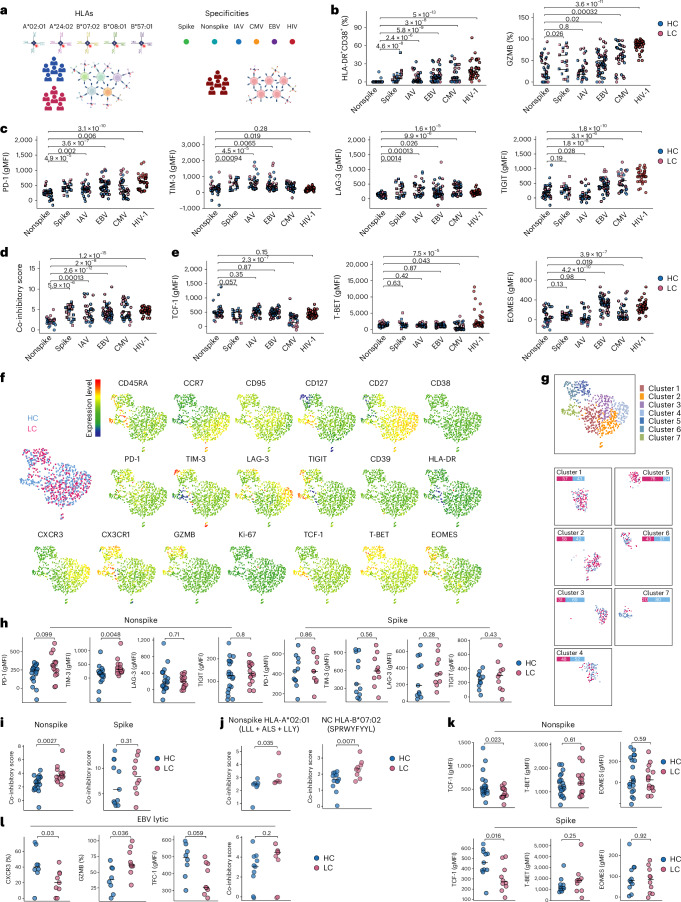
Phenotypic characteristics of virus-specific CD8^+^ T cells in healthy convalescent individuals and individuals with long COVID recruited from the United Kingdom. **a**, Schematic representation of the experimental design. **b**, Scatter dot plots showing the expression frequencies of HLA-DR and CD38 or granzyme B (GZMB) among tetramer^+^CD8^+^ T cells. **c**, Scatter dot plots showing the expression intensities of co-inhibitory receptors among tetramer^+^CD8^+^ T cells. **d**, Scatter dot plot showing co-inhibitory scores, calculated as the cumulative normalized expression intensities of the co-inhibitory receptors shown in **c**, among tetramer^+^CD8^+^ T cells. **e**, Scatter dot plots showing the expression intensities of transcription factors among tetramer^+^CD8^+^ T cells. **f**, UMAP visualization summarizing the phenotypic characteristics of tetramer^+^CD8^+^ T cells targeting nonspike epitopes from SARS-CoV-2. Individual marker representations are colored by expression intensity. **g**, Phenograph clustering (top) and cluster distribution of tetramer^+^CD8^+^ T cells (bottom). **h**, Scatter dot plots showing the expression intensities of co-inhibitory receptors among tetramer^+^CD8^+^ T cells targeting nonspike or spike epitopes from SARS-CoV-2. **i**, Scatter dot plots showing co-inhibitory scores among tetramer^+^CD8^+^ T cells targeting nonspike or spike epitopes from SARS-CoV-2. **j**, Scatter dot plots showing co-inhibitory scores among tetramer^+^CD8^+^ T cells targeting nonspike epitopes from SARS-CoV-2 restricted by HLA-A*02:01 or HLA-B*07:02; NC, nucleocapsid. **k**, Scatter dot plots showing the expression intensities of transcription factors among tetramer^+^CD8^+^ T cells targeting nonspike or spike epitopes from SARS-CoV-2. **l**, Scatter dot plots showing the phenotypic characteristics of tetramer^+^CD8^+^ T cells targeting lytic epitopes from EBV; healthy convalescent individuals, *n* = 17; individuals with long COVID, *n* = 15; untreated individuals infected with HIV-1, *n* = 14 (**b**–**l**). Horizontal bars represent median values (**b**–**e** and **h**–**l**). Significance was evaluated using a two-tailed Mann–Whitney *U*-test (**b**–**e** and **h**–**l**); gMFI, geometric mean fluorescence intensity.

To refine our phenotypic analyses, which were potentially confounded by alterations in surface marker expression arising as a consequence of antigen-induced activation, we used peptide–HLA class I tetramers directly ex vivo to identify and characterize unperturbed CD8^+^ T cells targeting specific epitopes from SARS-CoV-2, CMV, EBV or influenza A virus (IAV)^25,26^. For this purpose, we selected healthy convalescent individuals (*n* = 17) and individuals with long COVID (*n* = 15) from the primary cohort based on the expression of HLA-A*02:01 and/or HLA-B*07:02. As a means to calibrate our findings against CD8^+^ T cells with known features of exhaustion^27^, we also performed similar analyses using samples from untreated individuals infected with human immunodeficiency virus type 1 (HIV-1), extending the range of specificities to include epitopes restricted by HLA-A*24:02, HLA-B*08:01 and HLA-B*57:01 (Fig. 4a).

**Extended Data Fig. 4:**
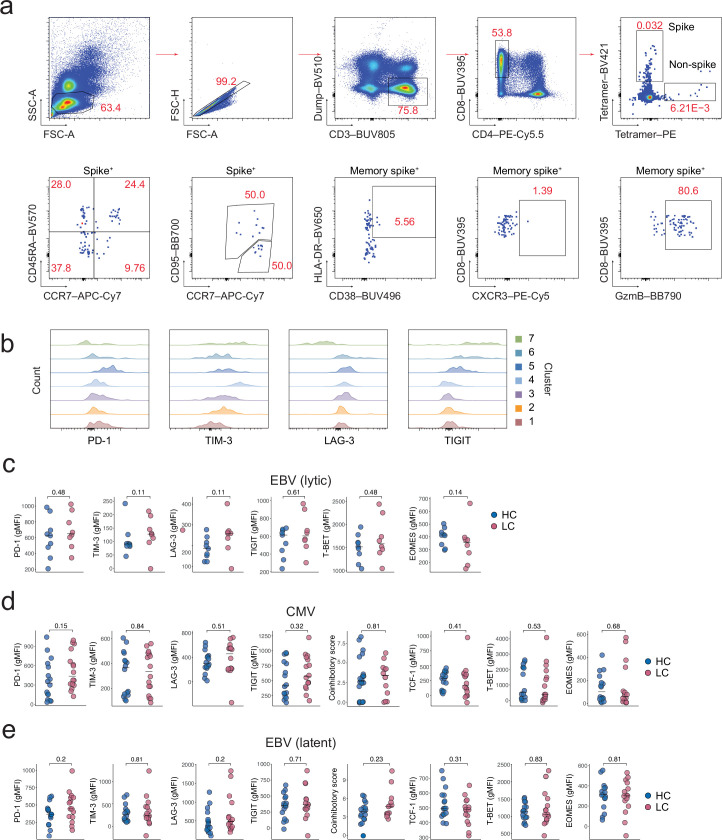
Additional phenotypic characteristics of virus-specific CD8^+^ T cells in healthy convalescent individuals and patients with long COVID recruited from the UK. (**a**) Flow cytometric gating strategy for the identification of tetramer^+^ CD8^+^ T cells directly *ex vivo*. Numbers indicate percentages in the drawn gates. (**b**) Flow cytometry histograms showing the expression patterns of coinhibitory receptors among clusters of tetramer^+^ CD8^+^ T cells targeting nonspike epitopes from SARS-CoV-2 identified using Phenograph. (**c**) Scatter dot plots showing the expression intensities of selected markers among tetramer^+^ CD8^+^ T cells targeting lytic epitopes from EBV. (**d**) Scatter dot plots showing the expression intensities of selected markers among tetramer^+^ CD8^+^ T cells targeting epitopes from CMV. (**e**) Scatter dot plots showing the expression intensities of selected markers among tetramer^+^ CD8^+^ T cells targeting latent epitopes from EBV. Healthy convalescent individuals (HC), *n* = 17; patients with long COVID (LC), *n* = 15 (**b**, **c**, **d** and **e**). Horizontal bars represent median values (**c**, **d** and **e**). Significance was evaluated using a two-tailed Mann–Whitney U test (**c**, **d** and **e**). gMFI, geometric mean fluorescence intensity.

CD8^+^ T cells targeting spike epitopes from SARS-CoV-2 expressed CD38 and HLA-DR more frequently than CD8^+^ T cells targeting nonspike epitopes from SARS-CoV-2 (Fig. 4b and Extended Data Fig. 4a), likely as a consequence of repeated subunit vaccination. Similarly, CD8^+^ T cells targeting viral epitopes associated with persistent (CMV, EBV and HIV-1) or recurrent antigen exposure (IAV) expressed CD38 and HLA-DR more frequently than CD8^+^ T cells targeting nonspike epitopes from SARS-CoV-2, and a comparable pattern was observed for expression of the cytotoxic serine protease granzyme B (Fig. 4b). Co-inhibitory receptor expression also varied as a function of viral specificity, typically paralleling the likely frequency of antigen exposure (Fig. 4c). Of particular note, we found that CD8^+^ T cells targeting spike epitopes from SARS-CoV-2 expressed co-inhibitory receptors more intensely than CD8^+^ T cells targeting nonspike epitopes from SARS-CoV-2, based on a combined score for PD-1, TIM-3, LAG-3 and TIGIT (Fig. 4d). No such differences were observed for the transcription factors TCF-1, T-BET or EOMES (Fig. 4e). However, CD8^+^ T cells targeting epitopes from CMV or HIV-1 expressed T-BET more intensely than CD8^+^ T cells targeting nonspike epitopes from SARS-CoV-2, and CD8^+^ T cells targeting epitopes from CMV, EBV or HIV-1 expressed EOMES more intensely than CD8^+^ T cells targeting nonspike epitopes from SARS-CoV-2 (Fig. 4e).

Collectively, these observations support the premise that antigen exposure drives the expression of activation markers and co-inhibitory receptors as a function of viral specificity and further suggest that such encounters are not sufficiently frequent in the convalescent phase to induce exhaustion among CD8^+^ T cells targeting nonspike epitopes from SARS-CoV-2, irrespective of progression to long COVID.

### SARS-CoV-2-specific CD8^+^ T cell phenotypes in long COVID

To determine if any of these phenotypic attributes segregated with disease, we visualized our flow cytometry data using the dimensionality reduction technique uniform manifold approximation and projection (UMAP), focusing on CD8^+^ T cells targeting nonspike epitopes from SARS-CoV-2. A largely overlapping distribution was observed for healthy convalescent individuals and individuals with long COVID (Fig. 4f). Phenograph analysis further revealed seven clusters, most of which displayed an even representation (Fig. 4g). However, clusters 3 and 7 were more obviously represented among healthy convalescent individuals, and cluster 5 was more obviously represented among individuals with long COVID (Fig. 4g). Of note, cluster 5 exhibited the highest expression intensities of co-inhibitory receptors, including PD-1 (Fig. 4g and Extended Data Fig. 4b). In line with this observation, we found that CD8^+^ T cells targeting nonspike epitopes from SARS-CoV-2 expressed co-inhibitory receptors more intensely in individuals with long COVID than in healthy convalescent individuals, reaching significance for TIM-3 (Fig. 4h). No such differences were observed for CD8^+^ T cells targeting spike epitopes from SARS-CoV-2 (Fig. 4h). Moreover, CD8^+^ T cells targeting nonspike epitopes from SARS-CoV-2 displayed higher co-inhibitory scores in individuals with long COVID than in healthy convalescent individuals, suggesting a link between antigen exposure and disease (Fig. 4i). It was also notable that co-inhibitory scores varied across specificities within the nonspike repertoire (Fig. 4j).

In further analyses, we found that CD8^+^ T cells targeting nonspike or spike epitopes from SARS-CoV-2 expressed TCF-1, a key determinant of memory formation, more intensely in healthy convalescent individuals than in individuals with long COVID (Fig. 4k). Moreover, CD8^+^ T cells targeting lytic epitopes from EBV expressed CXCR3 more frequently, granzyme B less frequently, and TCF-1 more intensely in healthy convalescent individuals than in individuals with long COVID (Fig. 4l and Extended Data Fig. 4c). No such differences were observed for CD8^+^ T cells targeting epitopes from CMV or CD8^+^ T cells targeting latent epitopes from EBV (Extended Data Fig. 4d,e).

Collectively, these findings suggest a possible role for cumulative viral antigen exposure in the pathogenesis of long COVID, potentially accompanied by suboptimal immune control of EBV.

### Plasma proteomic signatures of breathlessness in long COVID

**Fig. 5:**
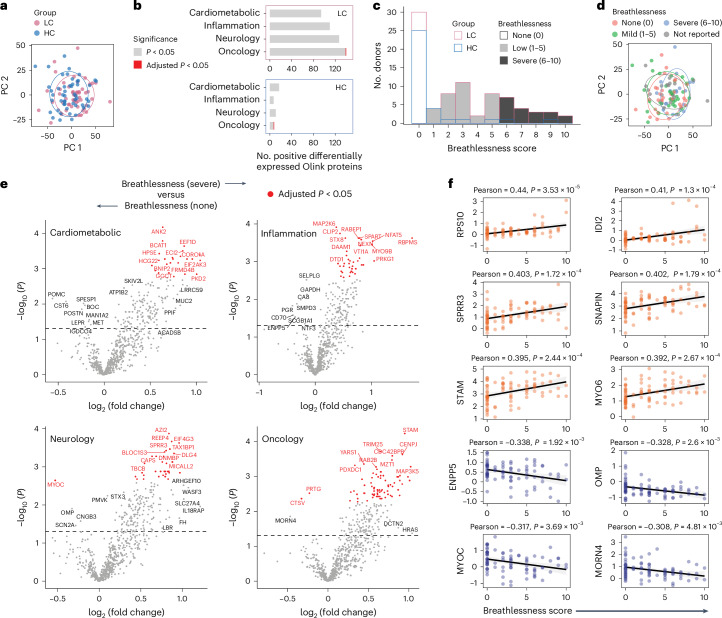
Dysregulation of the plasma proteome associated with breathlessness in healthy convalescent individuals and individuals with long COVID recruited from the United Kingdom. **a**, PCA of plasma protein concentrations colored by donor group for healthy convalescent individuals (*n* = 51) and individuals with long COVID (*n* = 51). **b**, Bar plots showing the corresponding numbers of differentially upregulated plasma proteins from each panel. Significance was evaluated using a two-tailed Mann–Whitney *U*-test with (red) or without (gray) Benjamini–Hochberg correction. **c**, Stacked histogram showing the distribution of breathlessness scores for healthy convalescent individuals (*n* = 34) and individuals with long COVID (*n* = 48). **d**, PCA of plasma protein concentrations colored by breathlessness score tiers for healthy convalescent individuals (*n* = 51) and individuals with long COVID (*n* = 51), irrespective of clinical assignation. **e**, Volcano plots showing the corresponding differentially expressed plasma proteins from each panel versus the highest and lowest breathlessness score tiers, irrespective of clinical assignation. Significance was evaluated using a two-tailed Mann–Whitney *U*-test with (red) or without (gray) Benjamini–Hochberg correction. The dashed line indicates *P* = 0.05. **f**, Correlation dot plots showing the highest (*n* = 6) and lowest ranked plasma proteins (*n* = 4) in terms of normalized expression versus breathlessness scores for healthy convalescent individuals (*n* = 51) and individuals with long COVID (*n* = 51), irrespective of clinical assignation; IDI2, isopentenyl-diphosphate δ-isomerase-2; SPRR3, small proline-rich protein 3; ENPP5, ectonucleotide pyrophosphatase/phosphodiesterase family member 5; OMP, olfactory marker protein. Significance was evaluated using the two-tailed Pearson coefficient. Shading indicates the 95% confidence interval for each regression line.

**Extended Data Fig. 5:**
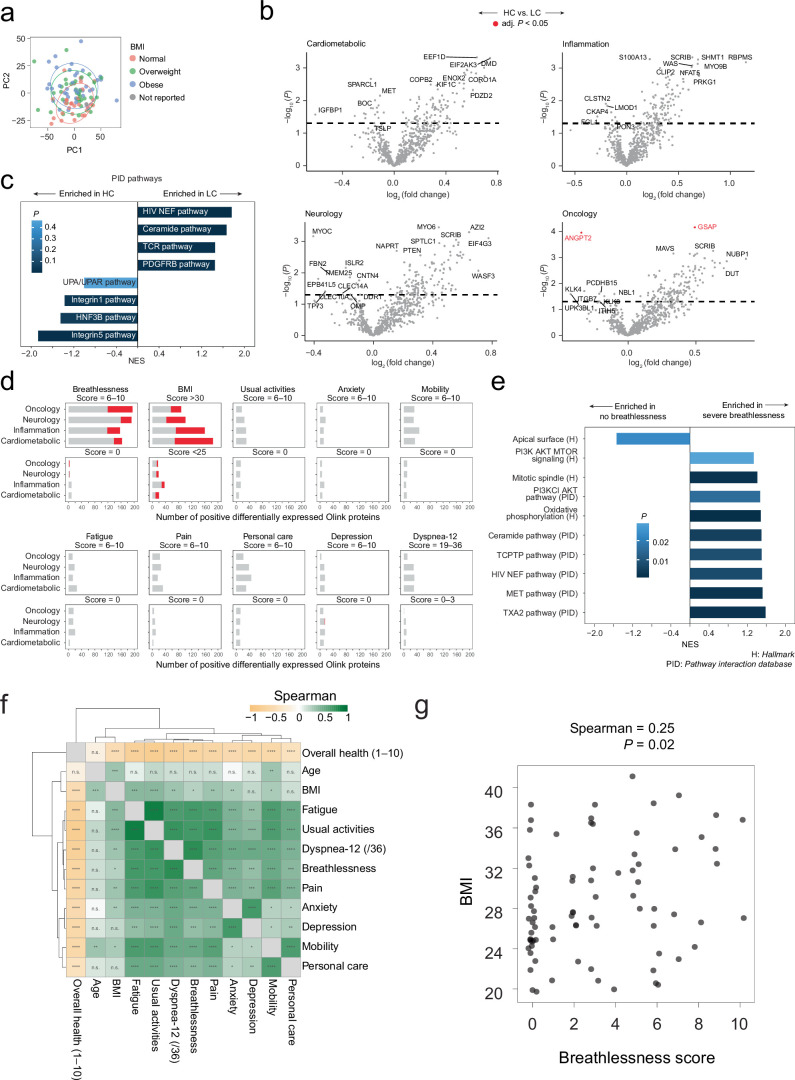
Dysregulation of the plasma proteome associated with clinical assignation and symptomatology in healthy convalescent individuals and patients with long COVID recruited from the UK. (**a**) Principal component analysis of plasma protein concentrations colored by body mass index (BMI) for healthy convalescent individuals (*n* = 51) and patients with long COVID (*n* = 51). (**b**) Volcano plots showing the corresponding differentially expressed plasma proteins from each panel versus clinical assignation. Significance was evaluated using a two-tailed Mann–Whitney U test with (red) or without Benjamini-Hochberg correction (gray). The dashed line indicates *P* = 0.05. (**c**) Gene set enrichment analysis (GSEA) showing the corresponding differentially expressed plasma proteins by rank versus clinical assignation. (**d**) Bar plots showing the numbers of differentially upregulated plasma proteins from each panel versus the highest and lowest symptom score tiers for healthy convalescent individuals (*n* = 34) and patients with long COVID (*n* = 48), irrespective of clinical assignation. Significance was evaluated using a two-tailed Mann–Whitney U test with (red) or without Benjamini-Hochberg correction (gray). (**e**) GSEA showing the corresponding differentially expressed plasma proteins by rank versus the highest and lowest breathlessness score tiers, irrespective of clinical assignation. (**f**) Heatmap showing correlations among clinical scores for healthy convalescent individuals (*n* = 51) and patients with long COVID (*n* = 51), irrespective of clinical assignation. (**g**) Correlation dot plot showing the corresponding breathlessness scores versus BMI, irrespective of clinical assignation. Significance was evaluated using the GSEA method without correction (**c** and **e**) or a two-tailed Spearman rank test (**f** and **g**). H, Hallmark; NES, normalized enrichment score; PID, Pathway Interaction Database.

To explore disease pathogenesis more systematically, we used a data-driven approach to select healthy convalescent individuals (*n* = 51) and individuals with long COVID (*n* = 51) from the primary cohort for plasma proteome characterization using a Proximity Extension Assay (Olink Explore 3072). Briefly, immune cell subset proportions were summarized via principal component analysis (PCA), and outlier samples were excluded based on the greatest deviation from the origin along PC1 to PC4. Target proteins were grouped into eight panels under the following broad themes: cardiometabolic (*n* = 2), inflammation (*n* = 2), neurology (*n* = 2) and oncology (*n* = 2). PCA revealed that donors could not be separated by disease status (Fig. 5a) but could be separated to some extent by BMI (Extended Data Fig. 5a). We then performed a differential expression analysis, which revealed a skewed upregulation of many proteins in individuals with long COVID (Supplementary Table 2), although most fell below the threshold for significance after multiple-hypothesis correction (Fig. 5b and Extended Data Fig. 5b). A gene set enrichment analysis (GSEA) further showed that several pathways, including those related to ceramide, platelet-derived growth factor receptor-β (PDGFRB) and HIV-1 Nef, were associated with this proteomic signature of long COVID (Extended Data Fig. 5c).

To extend our analyses beyond a simple binary classification, we stratified donors into three groups for each clinical symptom, irrespective of the initial categorization as healthy convalescent individuals or individuals with long COVID (Fig. 5c and Extended Data Fig. 5d). Using this approach, we found that breathlessness was strongly associated with differential protein expression (Extended Data Fig. 5d). Donors with severe breathlessness (score of 6–10) segregated from donors with no (score of 0) or mild breathlessness (score of 1–5) via PCA (Fig. 5d) and exhibited distinct patterns of protein upregulation (Fig. 5e and Supplementary Table 3). Moreover, GSEA confirmed that severe breathlessness was associated with the enrichment of several pathways that characterized the proteomic signature of long COVID, including those related to ceramide and HIV-1 Nef (Extended Data Fig. 5e). Of note, breathlessness and other symptom scores were largely independent of age and correlated only weakly with BMI (Extended Data Fig. 5f,g), which is known to impact the plasma proteome^28^.

**Extended Data Fig. 6:**
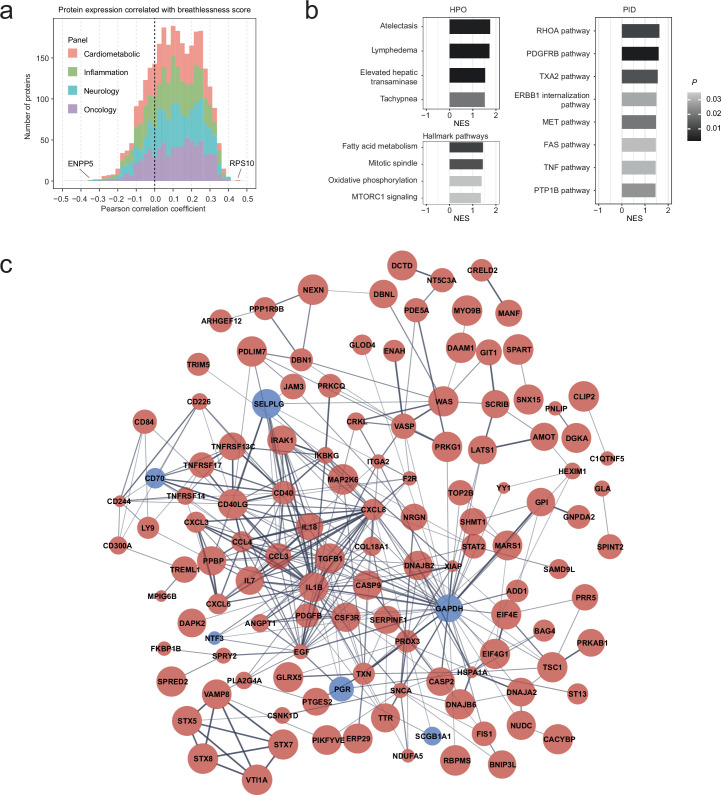
Dysregulation of the plasma proteome associated with breathlessness in healthy convalescent individuals and patients with long COVID recruited from the UK. (**a**) Stacked histogram showing the distribution of correlation coefficients from pairwise comparisons of plasma protein concentrations versus breathlessness scores for healthy convalescent individuals (*n* = 34) and patients with long COVID (*n* = 48), irrespective of clinical assignation. Significance was evaluated using the two-tailed Pearson coefficient. (**b**) Gene set enrichment analysis (GSEA) showing selected terms from Human Phenotype Ontology (HPO), the Pathway Interaction Database (PID), and the Hallmark Collection. Plasma protein concentrations were ranked by correlation with breathlessness scores for healthy convalescent individuals (*n* = 34) and patients with long COVID (*n* = 48), irrespective of clinical assignation. Significance was evaluated using the GSEA method without correction. NES, normalized enrichment score. (**c**) Network analysis showing the corresponding differentially expressed plasma proteins from the inflammation panel depicted using Cytoscape. Each node represents a protein. Node color indicates protein concentration, and node size indicates significance. Red denotes overexpression in patients with long COVID, and blue denotes underexpression in patients with long COVID. Each edge represents the functional relevance between a pair of proteins, and line thickness represents the level of confidence.

To identify specific proteins associated with breathlessness, we performed a correlation analysis without prior stratification based on symptom severity. The concentrations of almost all plasma proteins were skewed toward a positive correlation with breathlessness score (Extended Data Fig. 6a and Supplementary Table 4). The most positively correlated proteins included isopentenyl-diphosphate δ-isomerase 2 and small proline-rich protein 3, and the most negatively correlated proteins included ectonucleotide pyrophosphatase/phosphodiesterase family member 5 and olfactory marker protein (Fig. 5f). We then used the list of proteins ranked according to correlation with breathlessness to perform a GSEA, which showed that dysregulation of the plasma proteome was associated with related phenotypes, such as atelectasis (lung collapse) and tachypnea (rapid breathing), and further revealed an enrichment for pathways linked to cell cycle progression (for example, RhoA), inflammation (for example, TNF) and platelet activation (for example, PDGFRB and thromboxane A2 (TXA2); Extended Data Fig. 6b). In a further step designed to identify proteins with outsized roles in the breathlessness signatures associated with inflammation, we performed network analyses using Cytoscape. The output highlighted a complex protein network centered around CD40 in a module that also included CCL3, CCL4, IKBKG and IL-18 (Extended Data Fig. 6c).

**Fig. 6:**
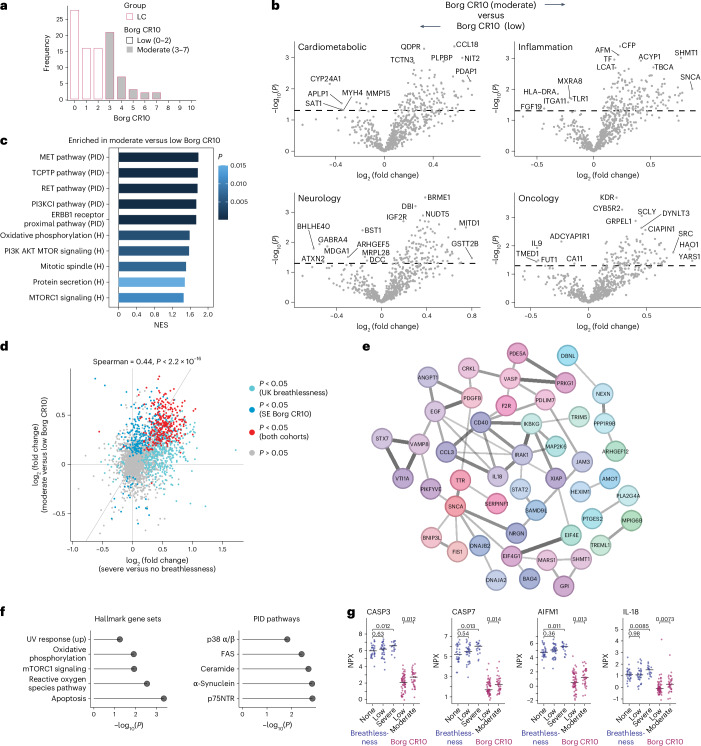
Shared signatures of plasma proteome dysregulation associated with respiratory symptoms in healthy convalescent individuals and individuals with long COVID. **a**, Bar plot showing the distribution of Borg CR10 scores for individuals in the secondary cohort with long COVID (*n* = 95). **b**, Volcano plots showing differentially expressed plasma proteins from each panel versus Borg CR10 score tiers for individuals in the secondary cohort with long COVID (*n* = 95). Significance was evaluated using a two-tailed Mann–Whitney *U-*test. The dashed line indicates *P* = 0.05. No proteins achieved adjusted *P* < 0.05 after Benjamini–Hochberg correction. **c**, GSEA showing differentially expressed plasma proteins by rank versus the Borg CR10 score tiers for individuals with long COVID (*n* = 95), irrespective of clinical assignation. The top five terms from the Hallmark (H) and Pathway Interaction Database (PID) gene sets (Molecular Signatures Database (MSigDB) Collections) are shown. Significance was evaluated using the GSEA method without correction; NES, normalized enrichment score. **d**, Scatter dot plot showing individual plasma protein fold change across breathlessness score tiers (primary cohort, *x* axis) and Borg CR10 score tiers (secondary cohort, *y* axis). Significance was evaluated using a two-tailed Spearman rank test. Proteins are colored according to significance without Benjamini–Hochberg correction. **e**, Network analysis showing differentially expressed plasma proteins from the inflammation panel across both cohorts depicted using Cytoscape. Nodes and edges represent proteins and functional relevance, respectively. Edge thickness represents the level of confidence. **f**, Overrepresentation analysis of significantly upregulated plasma proteins across both cohorts showing the five top terms from the Hallmark Collection and the Pathway Interaction Database. Significance was evaluated using a hypergeometric test. **g**, Comparison of plasma protein concentrations between cohorts split by symptom severity. Horizontal bars represent median values. Significance was evaluated using a two-tailed Mann–Whitney *U*-test; NPX, normalized protein expression; AIFM1, apoptosis-inducing factor mitochondria-associated 1; CASP, caspase.

**Extended Data Fig. 7:**
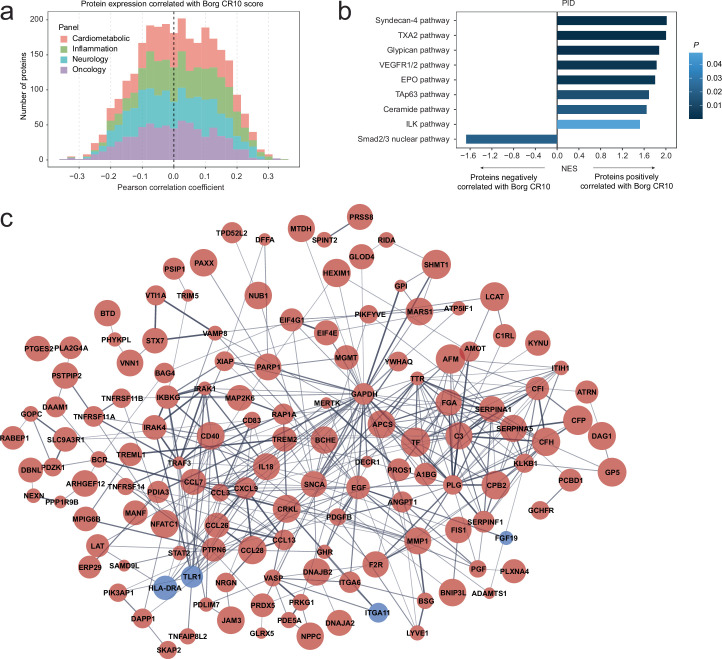
Dysregulation of the plasma proteome associated with perceived exertion in patients with long COVID recruited from Sweden. (**a**) Stacked histogram showing the distribution of correlation coefficients from pairwise comparisons of plasma protein concentrations versus Borg CR10 score for patients with long COVID (*n* = 95). Significance was evaluated using the two-tailed Pearson coefficient. (**b**) Gene set enrichment analysis (GSEA) showing plasma protein concentrations ranked by correlation with Borg CR10 scores for patients with long COVID (*n* = 95). Significance was evaluated using the GSEA method without correction. NES, normalized enrichment score. (**c**) Network analysis showing the corresponding differentially expressed plasma proteins from the inflammation panel depicted using Cytoscape. Each node represents a protein. Node color indicates protein concentration, and node size indicates significance. Red denotes overexpression in patients with a moderate Borg CR10 score, and blue denotes underexpression in patients with a moderate Borg CR10 score. Each edge represents the functional relevance between a pair of proteins, and line thickness represents the level of confidence.

To validate these findings, we performed similar analyses using plasma samples from donors in the secondary cohort, focusing on individuals with long COVID classified as low (0–2; *n* = 60) or moderate (3–7; *n* = 35) according to the Borg CR10 scale, which measures perceived exertion during physical activity (Fig. 6a)^29^. Differential expression analysis revealed upregulated protein expression among individuals with a moderate score, although no markers achieved significance after multiple-hypothesis correction (Fig. 6b and Supplementary Table 5). GSEA of the ranked list of proteins nonetheless identified enrichment of signaling pathways observed in the primary cohort (Extended Data Fig. 5e), including MET and PI3K/AKT/MTOR (Fig. 6c). Unbiased analyses further revealed that high Borg CR10 scores were correlated with pathways enriched among individuals in the primary cohort with severe breathlessness, including those associated with ceramide, syndecan-4 and TXA2 (Extended Data Fig. 7a,b and Supplementary Table 6).

To unify these data, we correlated protein expression across the primary and secondary cohorts as a function of symptom severity. A total of 275 proteins were differentially expressed among individuals with severe breathlessness and individuals with a moderate Borg CR10 score (Fig. 6d and Supplementary Table 7). Of these, all but three were consistently upregulated in both cohorts among donors with greater symptom severity, suggesting a shared signature of plasma proteome dysregulation in individuals with long COVID. Network analysis of differentially expressed inflammatory markers in the secondary cohort further identified a major hub centered around CD40 (Extended Data Fig. 7c), reminiscent of the primary cohort pattern (Extended Data Fig. 6c). A similar analysis of significantly upregulated proteins from the inflammation panel (*n* = 82) spanning both cohorts revealed that the most confident network connections were centered around CCL3, CD40, IKBKG, IL-18 and IRAK1 (Fig. 6e). Many of these proteins are associated with the NF-κB pathway. In addition, overrepresentation analysis of upregulated proteins spanning all panels across both cohorts identified apoptosis as the most significant hit among all gene sets in the Hallmark Collection and ceramide and FAS as highly significant hits in the Pathway Interaction Database (Fig. 6f). Of note, proteins associated with these pathways, including apoptosis-inducing factor mitochondria-associated 1, caspase-3, caspase-7 and IL-18, were specifically upregulated among donors with severe breathlessness recruited from the United Kingdom (Fig. 6g).

Collectively, these results identify dysregulated plasma proteins that could serve as biomarkers of persistent breathlessness after infection with SARS-CoV-2, potentially facilitating the diagnosis and treatment of long COVID.

## Discussion

Long COVID continues to pose medical challenges with unmet diagnostic and therapeutic needs that reflect the elusive mechanistic nature of a symptomatically heterogeneous disease. In this study, we used high-dimensional flow cytometry and plasma proteomics to seek biomarkers that could inform the pathogenesis of long COVID. Quantitative differences in immune cell lineage composition and virus-specific CD4^+^ and CD8^+^ T cell immunity were minimal and nonreproducible across two geographically distinct cohorts in direct comparisons of healthy convalescent individuals and individuals with long COVID. Antibody neutralization activity was nonetheless significantly higher in healthy convalescent individuals than in individuals with long COVID, despite comparable SARS-CoV-2 spike-specific IgG titers and equivalent levels of ADNKA, and some co-inhibitory receptors, especially PD-1 and TIM-3, were relatively overexpressed among SARS-CoV-2 nonspike-specific CD8^+^ T cells in individuals with long COVID. Our data also revealed an informative plasma biomarker signature linking persistent respiratory symptoms with apoptotic inflammatory networks and pathway dysregulation indicative of cell cycle progression, lung injury and platelet activation in individuals with long COVID.

Donor groups in our primary cohort were carefully matched for age, BMI, race, sex, time since infection, and vaccination against SARS-CoV-2, thereby minimizing the impact of confounding factors that could potentially bias comparative analyses of healthy convalescent individuals and individuals with long COVID. Women were overrepresented as a consequence^30–32^. The predominant symptoms were breathlessness, fatigue, pain, mobility issues, anxiety and depression, which align with the known clinical spectrum of long COVID^3^. Pain was localized primarily to the chest, joints and muscles, again consistent with distributions reported in other individuals with long COVID^33,34^. In contrast to influenza virus and other acute respiratory pathogens, which predominantly exacerbate localized symptoms during and after infection, these diverse postacute sequelae likely reflect an underlying etiological complexity, which mandates a systematic approach to the diagnosis and management of individuals with long COVID^35^.

In contrast to a recent study^16^, we found that healthy convalescent individuals were better able to neutralize SARS-CoV-2 than individuals with long COVID. This observation suggests a qualitative difference in antibody induction, potentially reflecting the fact that healthy convalescent individuals were vaccinated more frequently before infection than individuals with long COVID, which could help mitigate the risk of persistent disease^36^. No such differences were detected with respect to overall SARS-CoV-2 spike-specific IgG titers or ADNKA. This latter finding could be explained by the functional equivalence of antibodies targeting the spike protein^37^ and/or by the availability of nonspike targets expressed on the cell surface after infection with SARS-CoV-2 (ref. ^38^).

Systemic immune perturbations are thought to play a role in the pathogenesis of long COVID^3,21^. For example, innate immune cell activation and a paucity of naive B and T cells have been described in one cohort of individuals with long COVID^8^, whereas a relative abundance of highly cytotoxic CD8^+^ T cells and NK cells has been described in another cohort of individuals with long COVID^39^. We found only nuanced differences between the immune cell lineage profiles of healthy convalescent individuals and individuals with long COVID. In the primary cohort, these differences were limited to nonclassical monocytes, which were relatively overrepresented in healthy convalescent individuals, and basophils and pDCs, which were relatively overrepresented in individuals with long COVID, whereas in the secondary cohort, these differences were limited to classical monocytes, which were relatively overrepresented in healthy convalescent individuals, and intermediate monocytes, which were relatively overrepresented in individuals with long COVID. Such inconsistencies likely reflect a number of factors, including comorbidities and disease heterogeneity, and underscore the importance of cross-validation in studies of long COVID^3,10,40,41^.

SARS-CoV-2 proteins can be detected in many tissues long after the acute infectious event and could potentially engender a state of chronic immune activation linked with the development of long COVID^6,42,43^. It is also known that CD4^+^ and CD8^+^ memory T cells provide durable immunity against SARS-CoV-2 (refs. ^22,23,25,44^). These cells are exquisitely poised to mount anamnestic responses and would likely proliferate and shift to an activated and/or exhausted phenotype under conditions of recurrent antigen stimulation associated with a failure to clear residual viral products and/or ongoing viral replication, thereby feasibly becoming immunopathogenic rather than protective in the context of long COVID^10,45^. In line with this notion, one study reported sustained SARS-CoV-2-specific CD4^+^ T cell responses during late recovery in individuals with long COVID^46^, and another study reported enhanced expression of the exhaustion markers CTLA-4 and PD-1 among SARS-CoV-2-specific CD8^+^ T cells in individuals with long COVID^14^. The converse scenario in terms of response magnitude has also been described for IFNγ-producing CD8^+^ T cells targeting the nucleocapsid protein of SARS-CoV-2 (ref. ^47^). In our primary cohort, SARS-CoV-2-specific CD4^+^ and CD8^+^ T cell responses were comparable in magnitude across the entire viral proteome in healthy convalescent individuals and individuals with long COVID, whereas in our secondary cohort, relatively elevated frequencies of SARS-CoV-2 nucleocapsid-specific CD4^+^ T cells and SARS-CoV-2 spike-specific CD8^+^ T cells were observed in individuals with long COVID. However, more refined analyses of the primary cohort revealed altered memory profiles and enhanced co-inhibitory scores among SARS-CoV-2 nonspike-specific CD8^+^ T cells in individuals with long COVID, indicating a relatively greater cumulative history of exposure to antigens derived from SARS-CoV-2. An alternative possibility is that immune exhaustion facilitates viral persistence, but further studies are required to determine the protective versus reactive properties of SARS-CoV-2-specific CD4^+^ and CD8^+^ T cells in relation to the pathogenesis of long COVID.

Many factors can affect immune responses against SARS-CoV-2, including genetic background, infection history and vaccination status^7,48–50^, and many factors beyond immune responses against SARS-CoV-2 have been linked with the pathogenesis of long COVID^3,51^, including reactivation of the herpesviruses CMV and/or EBV^3,10,11,52,53^. Most of these latter associations have been defined serologically^16,20,54^. We addressed the same issue by interrogating CD4^+^ and CD8^+^ T cells targeting immunodominant regions of CMV or EBV. In the primary cohort, no intergroup differences in response magnitude were detected for any specificity, whereas in the secondary cohort, the frequencies of CD4^+^ T cells targeting EBV latent proteins and the frequencies of CD8^+^ T cells targeting CMV proteins or EBV lytic proteins were relatively elevated in individuals with long COVID. Phenotypic analyses focused on the primary cohort further revealed high co-inhibitory scores among CD8^+^ T cells targeting epitopes from CMV, which overexpressed PD-1, and a terminally differentiated profile among CD8^+^ T cells targeting lytic epitopes from EBV in individuals with long COVID. In line with these observations, we found that SARS-CoV-2 spike-specific CD8^+^ T cells overexpressed various activation markers, including CD38 and HLA-DR, and various co-inhibitory receptors spanning PD-1, TIM-3, LAG-3 and TIGIT, likely reflecting recurrent antigen exposure as a consequence of repeated subunit vaccination^55^. Accordingly, our data align with the notion that bystander viral reactivation frequently accompanies the development of persistent disease after infection with SARS-CoV-2 (refs. ^10,54,56^).

Plasma proteomics has emerged as a useful strategy to help decipher the molecular basis of various diseases via the identification of systemic biomarkers indicative of tissue-localized pathology^12,57–59^. Using a high-throughput platform in conjunction with a symptom-targeted approach, we found that severe breathlessness was associated with extensive dysregulation of the plasma proteome. It should be noted that our approach was focused on a curated panel of proteins spanning a targeted fraction of the entire proteome, such that we potentially failed to identify some biomarkers and pathways characteristic of long COVID. Our findings nonetheless align broadly with other strands of evidence indicating that chronic inflammation is a cornerstone of long COVID^8,12,14,15,60^. Moreover, network analyses identified connections centered around CD40, incorporating various caspases (CASP2 and CASP7) and kinases (IKBKG and MAP2K6), collectively linking breathlessness with inflammatory apoptosis and/or cell death, which could feasibly reflect ongoing exposure to antigens derived from SARS-CoV-2 (refs. ^61,62^). Similar inflammatory profiles have been identified previously in individuals with long COVID^12,13^. Pathway analyses further identified dysregulated proteins associated with cell cycle progression (for example, RhoA) and platelet activation (for example, PDGFRB and TXA2). In sum, these observations fit with a dynamic process of lung damage and remodeling attributable primarily to hypercoagulability and thromboinflammation^18^, potentially accompanied by amyloid fibrin microclot deposition^63^, endothelial dysfunction^64^ and vasculoproliferation^65^, which collectively impair oxygen exchange and lead to the sensation of breathlessness in individuals with long COVID.

One limitation of our study was that the matching process for the primary cohort was not entirely accurate, such that healthy convalescent individuals were sampled earlier after infection (median = 268 days) than individuals with long COVID (median = 416 days). This discrepancy combined with a preferential loss of functionally optimal antibodies could partially explain the relative paucity of neutralization activity in individuals with long COVID. A minority of healthy convalescent individuals also reported breathlessness as a symptom, likely attributable to other pathologies affecting the respiratory system, which were not assessed clinically. Moreover, our approach was limited to samples acquired from the vascular circulation, which emerging evidence suggests is a highly specialized immunological niche^66^. In addition, the origins and roles of proteins detected in plasma samples are open to interpretation, providing only indirect evidence for any given underlying pathology. Comparative analyses of disease-relevant tissue samples will therefore be required to validate the localized pathology associated with our reported systemic cellular and molecular signatures of long COVID.

In summary, our findings suggest that lung damage associated with the canonical symptom of breathlessness can be identified via the systemic upregulation of multiple apoptotic, cardiovascular and inflammatory biomarkers in the presence of a largely unperturbed cellular immune system, indicative of localized tissue pathology and ongoing but minimal exposure to viral antigens potentially facilitated by suboptimal humoral immunity in individuals with long COVID.

## Methods

### Study design

The objective of this study was to characterize the immunological and proteomic features of long COVID. SARS-CoV-2 spike-specific antibody titers were measured using an enzyme-linked immunosorbent assay. Antibody neutralization activity and ADNKA were quantified against the England-2 strain of SARS-CoV-2. Immune cell lineages were profiled via multidimensional flow cytometry. Antigen-specific CD4^+^ and CD8^+^ T cells were enumerated functionally using a flow cytometric AIM assay. Antigen-specific CD8^+^ T cells were further identified physically using peptide–HLA class I tetramers to enable detailed phenotypic analyses via multidimensional flow cytometry. Plasma proteomes were analyzed using a targeted affinity platform. Clinical symptoms were integrated with the frequencies and phenotypic attributes of immune cells to delineate plasma biomarkers and signaling pathways associated with long COVID.

### Donors

The primary cohort included healthy convalescent individuals (controls; *n* = 70) and individuals with long COVID (cases; *n* = 70) recruited from University Hospital Llandough (Table 1 and Supplementary Table 1). All participants had a clearly defined episode of symptomatically mild acute COVID-19 confirmed via direct molecular evidence of infection with SARS-CoV-2. None required hospitalization. Cases were diagnosed according to the National Institute for Health and Care Excellence guideline NG188 (https://www.nice.org.uk/guidance/ng188). Groups were matched as closely as possible for age, BMI, race, sex, time since infection, and vaccination against SARS-CoV-2 (Fig. 1a,b and Table 1). Eligible individuals were men and nonpregnant women over the age of 18 years with no alternative explanatory disease and symptoms that persisted for at least 12 weeks after the initial diagnosis of acute COVID-19. One persistent symptom was sufficient for the diagnosis of long COVID. All individuals underwent a comprehensive medical evaluation, including chest radiography, electrocardiography, lung function tests (spirometry with gas transfer as indicated and measurement of exhaled nitric oxide), and standard blood tests (autoantibody screens; bone, liver and kidney function; coagulation screens; full blood count; markers of nutrition). Symptoms were scored individually using a numeric self-rating scale from 0 (no symptom) to 10 (worst possible symptom). Overall general health was scored similarly on an inverse scale from 0 (worst possible) to 10 (best possible). The secondary cohort included healthy convalescent individuals (controls; *n* = 30) and individuals with long COVID (cases; *n* = 95) recruited from the Karolinska University Hospital (Table 2). All participants in the primary cohort were recruited between March and August 2022, and all participants in the secondary cohort were recruited between June and October 2022. PBMCs from donors with untreated chronic HIV-1 infection (*n* = 14) were obtained from the University of Alabama at Birmingham or the University of California, San Francisco.

### Samples

PBMCs were isolated via standard density gradient centrifugation and cryopreserved in fetal bovine serum (Thermo Fisher Scientific) containing 10% dimethyl sulfoxide (DMSO; Sigma-Aldrich). EDTA plasma samples were stored at −80 °C.

### Ethics

All participants provided written informed consent in accordance with the principles of the Declaration of Helsinki (2013). The primary study was approved by the Cardiff University School of Medicine Research Ethics Committee (21/55) and the Health Research Authority and Health and Care Research Wales (20/NW/0240), and the secondary study was approved by the Swedish Ethical Review Authority (2022-00100-01).

### Cells and viruses

A549 and VeroE6 cells expressing human angiotensin-converting enzyme 2 (ACE2) and transmembrane serine protease 2 (TMPRSS2) were used to support viral entry and propagation^67^. Antibody functionality assays were performed using the England-2 strain of SARS-CoV-2 (ref. ^38^).

### Peptides

SARS-CoV-2 peptides were manufactured as 15-mers overlapping by 11 amino acids spanning the spike protein (Peptides & Elephants) or as 20-mers overlapping by 10 amino acids spanning the nucleocapsid, combined membrane and envelope, ORF1a, ORF1b and ORF3–ORF10 proteins (Sigma-Aldrich). EBV peptides were manufactured as 15-mers overlapping by 11 amino acids spanning the BRLF1, BZLF1, BMLF1 and BARF1 proteins (lytic pool) and the EBNA1, EBNA2, EBNA3A, EBNA3B, EBNA3C and LMP2 proteins (latent pool; JPT Peptide Technologies). CMV peptides were manufactured as 15-mers overlapping by 11 amino acids spanning the combined IE-1, IE-2 and pp65 proteins (JPT Peptide Technologies). Lyophilized peptides were reconstituted at a stock concentration of 10 mg ml^–1^ in DMSO and further diluted to 100 μg ml^–1^ in phosphate-buffered saline (PBS).

### Tetramers

Peptide–HLA class I complexes were generated and tetramerized with fluorescent tags as described previously^68,69^. The following specificities were used in this study: CMV pp65 HLA-A*02:01 NLVPMVATV (BV421), CMV pp65 HLA-B*07:02 TPRVTGGGAM (PE), EBV BMLF1 (lytic) HLA-A*02:01 GLCTLVAML (PE), EBV EBNA3A (latent) HLA-B*07:02 RPPIFIRRL (BV421), HIV-1 p2p7p1p6 Gag HLA-A*02:01 FLGKIWPSHK (PE), HIV-1 p17 Gag HLA-A*02:01 SLYNTVATL (BV421), HIV-1 Pol HLA-A*02:01 ILKEPVHGV (PE), HIV-1 p17 Gag HLA-A*24:02 KYKLHIVW (BV421), HIV-1 Nef HLA-A*24:02 RYPLTFGW (PE), HIV-1 p24 Gag HLA-B*07:02 GPGHKARVL (BV421), HIV-1 p24 Gag HLA-B*08:01 EIYKRWII (PE), HIV-1 p24 Gag HLA-B*57:01 KAFSPEVIPMF (PE), HIV-1 p24 Gag HLA-B*57:01 QASQEVKNW (BV421), IAV matrix protein M1 HLA-A*02:01 GILGFVFTL (BV421), IAV nucleoprotein HLA-B*07:02 LPFDKTTVM (BV421), SARS-CoV-2 spike HLA-A*02:01 YLQPRTFLL (BV421), SARS-CoV-2 nucleocapsid HLA-A*02:01 LLLDRLNQL (PE), SARS-CoV-2 ORF3 HLA-A*02:01 ALSKGVHFV (PE), SARS-CoV-2 ORF3 HLA-A*02:01 LLYDANYFL (PE) and SARS-CoV-2 nucleocapsid HLA-B*07:02 SPRWYFYYL (PE).

### Antibody quantification

SARS-CoV-2 spike-specific antibody titers were measured using a SARS-CoV-2 Spike (Trimer) Ig Total ELISA Kit (Thermo Fisher Scientific). Samples were assayed in duplicate and calibrated against a standard curve. Data were analyzed using Prism version 9.5.0 (GraphPad).

### Neutralization assay

Antibody neutralization activity was quantified as described previously^38^. Briefly, serial dilutions of plasma were mixed in duplicate with 600 plaque-forming units of England-2, incubated for 1 h at 37 °C, and added to VeroE6 cells expressing ACE2 and TMPRSS2. After 48 h, cell monolayers were fixed in 4% paraformaldehyde (Thermo Fisher Scientific), permeabilized with 0.5% NP-40 (Merck), and blocked with PBS containing 0.1% Tween-20 (PBST) and 3% nonfat milk for 1 h at room temperature (RT). The primary antibody (anti-SARS-CoV-2 nucleocapsid protein, clone 1C7, Stratech Scientific) was diluted 1:500 in PBST containing 1% nonfat milk and added to the cell monolayers for 1 h at RT. Cells were then washed with PBST. The secondary antibody (anti-mouse IgG-HRP, polyclonal, Jackson ImmunoResearch) was diluted 1:3,000 in PBST containing 1% nonfat milk and added to the cell monolayers for 1 h at RT. Cells were then washed again with PBST. Assays were developed using SIGMAFAST OPD (Sigma-Aldrich) and analyzed at an optical density of 450 nm using a CLARIOstar Plus Microplate Reader (BMG Labtech). Control wells contained no sample, a standardized sample with moderate neutralization activity, or no SARS-CoV-2. The neutralization titer for each sample was calculated as the highest plasma dilution that achieved a 50% reduction in plaque formation (NT_50_).

### ADNKA

ADNKA was quantified as described previously^38,70^. Briefly, target A549 cells expressing ACE2 and TMPRSS2 were infected overnight with England-2 (multiplicity of infection = 5), collected using TrypLE Express Enzyme (Thermo Fisher Scientific), mixed with healthy donor PBMCs at a ratio of 1:10, and incubated with serial dilutions of plasma in the presence of anti-CD107a–FITC (clone H4A3, BioLegend) and GolgiStop (0.7 μl ml^–1^; BD Biosciences) for 5 h at 37 °C. Cells were then washed with cold PBS, stained with anti-CD3–PE-Cy7 (clone UCHT1, BioLegend), anti-CD56–BV605 (clone 5.1H11, BioLegend), anti-CD57–APC (clone HNK-1, BioLegend) and LIVE/DEAD Fixable Aqua (Thermo Fisher Scientific) for 30 min at 4 °C, washed again with cold PBS, and fixed in 4% paraformaldehyde (Thermo Fisher Scientific). Control wells contained a seronegative sample, uninfected target cells, or a standardized sample that elicited moderate ADNKA. Data were acquired using an Attune NxT Flow Cytometer (Thermo Fisher Scientific). Activation was quantified as a function of degranulation (CD107a^+^) among viable NK cells (Aqua^−^CD3^−^CD56^+^) with potent cytotoxic activity (CD57^+^) using FlowJo version 10.9.0 (FlowJo) and normalized to the standardized sample via area under the curve analyses in Prism version 9.5.0 (GraphPad).

### Immune cell lineage analysis

PBMCs were thawed quickly, resuspended in RPMI 1640 Complete Medium (Sigma-Aldrich) supplemented with DNase I (10 U ml^–1^; Sigma-Aldrich), and seeded at 1 × 10^6^ cells per well in 96-well U-bottom plates (Corning). Cells were incubated first with Human TruStain FcX (BioLegend) for 10 min at RT and then with LIVE/DEAD Fixable Aqua (Thermo Fisher Scientific) for 10 min at RT. Anti-CCR7–APC-Cy7 (clone G043H7, BioLegend) and anti-CX3CR1–PE (clone 2A9-1, BioLegend) were added for 15 min at 37 °C. Cells were then stained with anti-CD3–BV650 (clone OKT3, BioLegend), anti-CD4–PE-Cy5.5 (clone S3.5, Thermo Fisher Scientific), anti-CD8–BUV396 (clone RPA-T8, BD Biosciences), anti-CD11c–BB515 (clone B-ly6, BD Biosciences), anti-CD14–PE-Cy5 (clone 61D3, Thermo Fisher Scientific), anti-CD16–BUV496 (clone 3G8, BD Biosciences), anti-CD19–BUV563 (clone HIB19, BD Biosciences), anti-CD27–BV786 (clone O323, BioLegend), anti-CD34–BB660 (clone 581, BD Biosciences), anti-CD38–APC (clone HB7, BD Biosciences), anti-CD45–BUV805 (clone HI30, BD Biosciences), anti-CD45RA–BV570 (clone HI100, BioLegend), anti-CD56–BUV615 (clone NCAM16.2, BD Biosciences), anti-CD69–BUV737 (clone FN50, BD Biosciences), anti-CD71–BUV661 (clone M-A712, BD Biosciences), anti-CD83–BB790 (clone HB15e, BD Biosciences), anti-CD86–BB630 (clone 2331 (FUN-1), BD Biosciences), anti-CD123–PE-Cy7 (clone 7G3, BD Biosciences), anti-CD127–BV421 (clone A019D5, BioLegend), anti-HLA-DR–BV605 (clone G46-6, BD Biosciences) and anti-PD-1–R718 (clone EH12.1, BD Biosciences) for 30 min at RT (Supplementary Table 8). Stained cells were washed twice with FACS buffer (PBS containing 2% fetal bovine serum and 2 mM EDTA), fixed in Cytofix Fixation Buffer (BD Biosciences), and acquired using a FACSymphony A3 (BD Biosciences). Data were analyzed using FlowJo version 10.9.0 (FlowJo).

### AIM assay

PBMCs were thawed quickly, resuspended in RPMI 1640 Complete Medium (Sigma-Aldrich) supplemented with DNase I (10 U ml^–1^; Sigma-Aldrich), and rested at 1 × 10^6^ cells per well in 96-well U-bottom plates (Corning) for 3 h at 37 °C. The medium was then supplemented with unconjugated anti-CD40 (clone HB14, Miltenyi Biotec) and anti-CXCR5–BB515 (clone RF8B2, BD Biosciences), followed 15 min later by the relevant peptides (each at 0.5 μg ml^–1^), and the cultures were incubated for 12 h at 37 °C. Negative-control wells contained equivalent DMSO. After incubation, cells were washed with PBS, labeled with LIVE/DEAD Fixable Aqua (Thermo Fisher Scientific) for 10 min at RT, washed with FACS buffer, and stained with anti-CCR4–BB700 (clone 1G1, BD Biosciences), anti-CCR6–BUV737 (clone 11A9, BD Biosciences), anti-CCR7–APC-Cy7 (clone G043H7, BioLegend), anti-CX3CR1–PE (clone 2A9-1, BioLegend) and anti-CXCR3–AF647 (clone G025H7, BioLegend) for 10 min at 37 °C. Cells were then stained further with anti-CD3–BUV805 (clone UCHT1, BD Biosciences), anti-CD4–BUV496 (clone SK3, BD Biosciences), anti-CD8–BUV395 (clone RPA-T8, BD Biosciences), anti-CD14–BV510 (clone M5E2, BioLegend), anti-CD19–BV510 (clone HIB19, BioLegend), anti-CD28–BUV563 (clone CD28.2, BD Biosciences), anti-CD38–APC-R700 (clone HIT2, BD Biosciences), anti-CD39–BV711 (clone A1, BioLegend), anti-CD45RA–BV570 (clone HI100, BioLegend), anti-CD69–BV650 (clone FN50, BioLegend), anti-CD71–BUV661 (clone M-A712, BD Biosciences), anti-CD95–PE-Dazzle594 (clone DX2, BioLegend), anti-CD127–PE-Cy5 (clone A019D5, BioLegend), anti-CD137–PE-Cy7 (clone 4B4-1, BioLegend), anti-CD154–BV421 (clone 24-31, BioLegend), anti-HLA-DR–BV605 (clone G46-6, BD Biosciences), anti-PD-1–BUV615 (clone EH12.1, BD Biosciences) and anti-TIGIT–BV786 (clone 741182, BD Biosciences) for 30 min at RT in the presence of Brilliant Stain Buffer Plus (BD Biosciences; Supplementary Table 9). Stained cells were washed twice with FACS buffer, fixed in Cytofix Fixation Buffer (BD Biosciences), and acquired using a FACSymphony A5 (BD Biosciences). Data were analyzed using FlowJo version 10.9.0 (FlowJo).

### Tetramer staining and phenotypic analysis

PBMCs were thawed quickly, resuspended in RPMI 1640 Complete Medium (Sigma-Aldrich) supplemented with DNase I (10 U ml^–1^; Sigma-Aldrich), and seeded at 2 × 10^6^ cells per well in 96-well U-bottom plates (Corning). Cells were incubated first with dasatinib (50 µM; STEMCELL Technologies) for 10 min at RT and then with the relevant peptide–HLA class I tetramers (each at 1 µg per stain) for 20 min at RT (Supplementary Table 10). After incubation, cells were washed with PBS, labeled with LIVE/DEAD Fixable Aqua (Thermo Fisher Scientific) for 10 min at RT, washed with FACS buffer, and stained with anti-CCR7–APC-Cy7 (clone G043H7, BioLegend), anti-CX3CR1–BUV615 (clone 2A9-1, BD Biosciences) and anti-CXCR3–PE-Cy5 (clone G025H7, BioLegend) for 10 min at 37 °C. Cells were then stained further with anti-CD3–BUV805 (clone UCHT1, BD Biosciences), anti-CD4–PE-Cy5.5 (clone RM4-5, Thermo Fisher Scientific), anti-CD8–BUV395 (clone RPA-T8, BioLegend), anti-CD14–BV510 (clone M5E2, BioLegend), anti-CD19–BV510 (clone HIB19, BioLegend), anti-CD27–BV786 (clone O323, BioLegend), anti-CD38–BUV496 (clone HIT2, BD Biosciences), anti-CD39–BV711 (clone A1, BioLegend), anti-CD45RA–BV570 (clone HI100, BioLegend), anti-CD95–BB700 (clone DX2, BD Biosciences), anti-CD127–BB630 (clone HIL-7R-M21, BD Biosciences), anti-HLA-DR–BV650 (clone G46-6, BD Biosciences), anti-LAG-3–BUV661 (clone 3DS223H, Thermo Fisher Scientific), anti-PD-1–BUV737 (clone EH12.1, BD Biosciences), anti-TIGIT–PE-Dazzle594 (clone A15153G, BioLegend) and anti-TIM-3–BV605 (clone F38-2E2, BioLegend) for 20 min at RT, washed twice with FACS buffer, fixed/permeabilized using a FoxP3 Transcription Factor Staining Buffer Set (Thermo Fisher Scientific), and stained intracellularly with anti-EOMES–EF660 (clone WD1928, eBioscience), anti-granzyme B–BB790 (clone GB11, BD Biosciences), anti-Ki67–AF700 (clone B56, BD Biosciences), anti-T-BET–PE-Cy7 (clone 4B10, eBioscience) and anti-TCF-1–AF488 (clone C63D9, Cell Signaling Technology) for 30 min at RT (Supplementary Table 11). Stained cells were washed twice with FACS buffer and acquired using a FACSymphony A3 (BD Biosciences). Data were analyzed using FlowJo software version 10.9.0 (FlowJo).

### Plasma proteomics

A data-driven approach was used to select healthy convalescent individuals (*n* = 51) and individuals with long COVID (*n* = 51) for plasma proteome characterization via a Proximity Extension Assay (Olink Proteomics). Immune cell subset proportions were summarized using a PCA. Outlier samples were excluded based on the greatest deviation from the origin along PC1 to PC4. Plasma samples were analyzed in two batches using Explore 3072 (Olink Proteomics). Sixteen bridge samples were included for quality control purposes in each batch.

### General statistics

Differences between groups were assessed using a two-tailed Mann–Whitney *U*-test. Raw *P* values are shown. Correlations were evaluated using the two-tailed Pearson coefficient or a two-tailed Spearman rank test. Significance was assigned at *P* < 0.05. Basic statistical analyses were performed using Prism version 9.5.0 (GraphPad).

### Flow cytometry data analysis

Samples acquired for immune cell lineage analysis were gated to the single-cell/viable/CD45^+^ population and subsequently exported to contain only 3,000 events using the FlowJo Plugin DownSample version 3. Exported fcs files were loaded into R using flowCore version 2.6.0. All data were concatenated into a single matrix with compensated markers (excluding viability, CD34 and CD45). Data for each marker were scaled and centered for analysis using umap version 0.2.10.0. Clustering was performed using a Gaussian mixture model (maxNumComponents = 10) implemented in mclust version 6.0.0. Data were visualized using ggplot2 version 3.4.2. Antigen-specific CD4^+^ and CD8^+^ T cell frequencies assessed via the AIM assay were calculated after background subtraction. Samples acquired for detailed phenotypic characterization were excluded below a threshold of five tetramer^+^ CD8^+^ T cells per specificity. The expression of each marker was then normalized to the average geometric mean fluorescence intensity across all samples and specificities and used to calculate the co-inhibitory score, representing the summed data for PD-1, TIM-3, LAG-3 and TIGIT. Statistical analyses were performed using R version 4.2.1.

### Plasma proteome data analysis

Bridge sample data were normalized using the olink_normalization function implemented in OlinkAnalyze version 3.4.1. Differential expression analyses were performed using a Wilcoxon rank-sum/Mann–Whitney *U*-test with Benjamini–Hochberg correction implemented via the olink_wilcox function in OlinkAnalyze version 3.4.1. GSEA was performed using fgsea version 1.20.0 incorporating lists of all analyzed proteins ordered by correlation coefficient or fold change. Gene sets were downloaded from the MSigDB using msigdb version 7.5.1. Overrepresentation analysis was performed using the fora function implemented in fgsea version 1.20.0 incorporating all measured proteins as the ‘universe’. At least five proteins were required in each gene set for consideration. Significance was evaluated using a hypergeometric test. Correlations were calculated using the cor.test function implemented in stats version 4.1.3. PCAs were performed using the prcomp function implemented in stats version 4.1.3. Data were visualized using ggplot2 version 3.4.2 and pheatmap version 1.0.12. All analyses were performed using R version 4.2.1. Network analyses of plasma proteins that were differentially expressed as a function of symptom severity were performed using the stringApp in Cytoscape version 3.10.3 (ref. ^71^).

### Reporting summary

Further information on research design is available in the Nature Portfolio Reporting Summary linked to this article.

## Online content

Any methods, additional references, Nature Portfolio reporting summaries, source data, extended data, supplementary information, acknowledgements, peer review information, details of author contributions and competing interests, and statements of data and code availability are available at 10.1038/s41590-025-02135-5.

## Supplementary information

Reporting Summary

Supplementary Tables 1–11 Supplementary Table 1. Cohort information for participants recruited from the United Kingdom. Supplementary Table 2. Differentially expressed plasma proteins comparing cases and controls recruited from the United Kingdom. Supplementary Table 3. Differentially expressed plasma proteins comparing no breathlessness and severe breathlessness among participants recruited from the United Kingdom. Supplementary Table 4. Correlation values between plasma protein expression levels and breathlessness scores among participants recruited from the United Kingdom. Supplementary Table 5. Differentially expressed plasma proteins comparing low and moderate Borg CR10 scores among individuals with long COVID recruited from Sweden. Supplementary Table 6. Correlation values between plasma protein expression levels and Borg CR10 scores among individuals with long COVID recruited from Sweden. Supplementary Table 7. Combined differential plasma protein expression testing results based on comparisons of Borg CR10 (Sweden) and breathlessness scores (United Kingdom). Supplementary Table 8. List of antibodies used for immune cell lineage analysis via flow cytometry. Supplementary Table 9. List of antibodies used for the functional detection of virus-specific CD4^+^ and CD8^+^ T cells via flow cytometry. Supplementary Table 10. List of peptide–HLA class I tetramers used for the physical detection of virus-specific CD8^+^ T cells via flow cytometry. Supplementary Table 11. List of antibodies used in conjunction with peptide–HLA class I tetramers for the physical detection of virus-specific CD8^+^ T cells via flow cytometry.

## Data Availability

Raw proteomics data are available via Zenodo (10.5281/zenodo.14772494)^72^. Any additional information required to reanalyze the data reported in this paper is available from the corresponding authors upon reasonable request.
