# Supplementary material for: Identification of soluble biomarkers that associate with distinct manifestations of long COVID
Source: Nat Immunol. 2025 Apr 30;26(5):692–705. doi: 10.1038/s41590-025-02135-5 (PMC12043503; doi:10.1038/s41590-025-02135-5)
Supplement: Supplementary file 1 — Reporting Summary [file 41590_2025_2135_MOESM1_ESM.pdf]

nature portfolio

Corresponding author(s): Marcus Buggert, David A Price

Last updated by author(s): Feb 26, 2025

Reporting Summary

Nature Portfolio wishes to improve the reproducibility of the work that we publish. This form provides structure for consistency and transparency in reporting. For further information on Nature Portfolio policies, see our Editorial Policies and the Editorial Policy Checklist.

Statistics

For all statistical analyses, confirm that the following items are present in the figure legend, table legend, main text, or Methods section.

n/a	Confirmed	
☐	☒	The exact sample size (*n*) for each experimental group/condition, given as a discrete number and unit of measurement
☐	☒	A statement on whether measurements were taken from distinct samples or whether the same sample was measured repeatedly
☐	☒	The statistical test(s) used AND whether they are one- or two-sided<br>*Only common tests should be described solely by name; describe more complex techniques in the Methods section.*
☒	☐	A description of all covariates tested
☒	☐	A description of any assumptions or corrections, such as tests of normality and adjustment for multiple comparisons
☒	☐	A full description of the statistical parameters including central tendency (e.g. means) or other basic estimates (e.g. regression coefficient) AND variation (e.g. standard deviation) or associated estimates of uncertainty (e.g. confidence intervals)
☒	☐	For null hypothesis testing, the test statistic (e.g. *F*, *t*, *r*) with confidence intervals, effect sizes, degrees of freedom and *P* value noted<br>*Give P values as exact values whenever suitable.*
☒	☐	For Bayesian analysis, information on the choice of priors and Markov chain Monte Carlo settings
☒	☐	For hierarchical and complex designs, identification of the appropriate level for tests and full reporting of outcomes
☐	☒	Estimates of effect sizes (e.g. Cohen's *d*, Pearson's *r*), indicating how they were calculated

Our web collection on statistics for biologists contains articles on many of the points above.

Software and code

Policy information about availability of computer code

Data collection	BD FACSDiva version 9.1<br>Olink Explore version 3.0.5
Data analysis	R version 4.2.1<br>FlowJo version 10.9.0<br>Prism version 10<br>CytoScape version 3.10.3

For manuscripts utilizing custom algorithms or software that are central to the research but not yet described in published literature, software must be made available to editors and reviewers. We strongly encourage code deposition in a community repository (e.g. GitHub). See the Nature Portfolio guidelines for submitting code & software for further information.

Data

Policy information about availability of data

All manuscripts must include a data availability statement. This statement should provide the following information, where applicable:

- Accession codes, unique identifiers, or web links for publicly available datasets
- A description of any restrictions on data availability
- For clinical datasets or third party data, please ensure that the statement adheres to our policy

Raw proteomics data are available via Zenodo (doi.org/10.5281/zenodo.14772494). This paper does not report original code. Data from the Molecular Signatures Database (MSigDB) used version 7.5.1. Any additional information required to reanalyze the data reported in this paper is available from the corresponding authors upon request.

Research involving human participants, their data, or biological material

Policy information about studies with human participants or human data. See also policy information about sex, gender (identity/presentation), and sexual orientation and race, ethnicity and racism.

Reporting on sex and gender	Biological sex is reported in Figure 1a, Table 1 and 2.
Reporting on race, ethnicity, or other socially relevant groupings	Ethnicity/race is reported in Table 1 and 2.
Population characteristics	Population characteristics are summarized Table 1 and 2.
Recruitment	Participants were recruited from two distinct sites as healthy convalescent individuals or patients with long COVID.
Ethics oversight	Cardiff University School of Medicine Research Ethics Committee<br>Health Research Authority and Health and Care Research Wales<br>Swedish Ethical Review Authority

Note that full information on the approval of the study protocol must also be provided in the manuscript.

Field-specific reporting

Please select the one below that is the best fit for your research. If you are not sure, read the appropriate sections before making your selection.

☒ Life sciences ☐ Behavioural & social sciences ☐ Ecological, evolutionary & environmental sciences

For a reference copy of the document with all sections, see nature.com/documents/nr-reporting-summary-flat.pdf

Life sciences study design

All studies must disclose on these points even when the disclosure is negative.

Sample size	No formal sample size calculation was performed. The sample size was determined based on feasibility, ethical considerations, and prior studies investigating similar research questions.
Data exclusions	No data were excluded from the flow cytometry analyses. A few samples were not selected for proteomic analyses due to cost limitations. Samples not selected for proteomic analyses were considered to be outliers based on circulating immune cell lineage composition (Figure 2).
Replication	Replication was not performed for individual samples due to limited material availability.
Randomization	Randomization was applied where relevant in this study. Specifically, for the OLINK Proteomics analysis, sample preparation followed the manufacturer's recommendations, which included a randomization step during sample loading onto the 96-well plate to minimize potential batch effects.
Blinding	Investigators were not blinded. All samples were treated identically. Each experimental protocol was performed identically for each sample.

Reporting for specific materials, systems and methods

We require information from authors about some types of materials, experimental systems and methods used in many studies. Here, indicate whether each material, system or method listed is relevant to your study. If you are not sure if a list item applies to your research, read the appropriate section before selecting a response.

Materials & experimental systems

n/a	Involved in the study
☐	☒ Antibodies
☐	☒ Eukaryotic cell lines
☒	☐ Palaeontology and archaeology
☒	☐ Animals and other organisms
☒	☐ Clinical data
☒	☐ Dual use research of concern
☒	☐ Plants

Methods

n/a	Involved in the study
☒	☐ ChIP-seq
☐	☒ Flow cytometry
☒	☐ MRI-based neuroimaging

Antibodies

Antibodies used

CCR7 APC-Cy7 353212 G043H7 BioLegend
CD38 APC 567144 HB7 BD Biosciences
PD-1 R718 566974 EH12.1 BD Biosciences
CD11c BB515 564491 B-ly6 BD Biosciences
CD86 BB630 Custom 2331(FUN-1) BD Biosciences
CD34 BB660 Custom 581 BD Biosciences
CD83 BB790 Custom HB15e BD Biosciences
CD16 BUV496 612945 3G8 BD Biosciences
CD19 BUV563 741361 HIB19 BD Biosciences
CD56 BUV615 613002 BD Biosciences
CD69 BUV737 612817 FN50 BD Biosciences
CD45 BUV805 612891 HI30 BD Biosciences
CD127 BV421 351310 A019D5 BioLegend
CD3 BV650 317323 OKT3 BioLegend
CD27 BV786 302832 O323 BioLegend
CD14 PE-Cy5 15-0149-41 61D3 Thermo Fisher Scientific
CD4 PE-Cy5.5 MHCD0418 S3.5 Thermo Fisher Scientific
CD123 PE-Cy7 560826 7G3 BD Biosciences
CD38 APC-R700 564979 HIT2 BD Biosciences
CXCR3 AF647 353712 G025H7 BioLegend
CCR4 BB700 566475 1G1 BD Biosciences
CXCR5 BB515 564624 RF8B2 BD Biosciences
CD137 PE-Cy7 309818 4B4-1 BioLegend
CD127 PE-Cy5 351324 A019D5 BioLegend
CD95 PE-Dazzle594 305634 DX2 BioLegend
CX3CR1 PE 341604 2A9-1 BioLegend
TIGIT BV786 747838 741182 BD Biosciences
CD39 BV711 328228 A1 BioLegend
CD69 BV650 310934 FN50 BioLegend
HLA-DR BV605 562845 G46-6 BD Biosciences
CD45RA BV570 304132 HI100 BioLegend
CD14 BV510 301842 M5E2 BioLegend
CD19 BV510 302242 HIB19 BioLegend
LIVE/DEAD Fixable Aqua L34966 Thermo Fisher Scientific
CD154 BV421 310824 24-31 BioLegend
CD3 BUV805 612895 UCHT1 BD Biosciences
CCR6 BUV737 612780 11A9 BD Biosciences
CD71 BUV661 750651 M-A712 BD Biosciences
PD-1 BUV615 612991 EH12.1 BD Biosciences
CD28 BUV563 741392 CD28.2 BD Biosciences
CD4 BUV496 612936 SK3 BD Biosciences
CD8 BUV395 563795 RPA-T8 BD Biosciences
CX3CR1 BUV615 751513 2A9-1 BD Biosciences
CXCR3 PE-Cy5 353756 G025H7 BioLegend
PD-1 BUV737 612791 EH12.1 BD Biosciences
LAG-3 BUV661 376-2239-42 3DS223H Thermo Fisher Scientific
CD38 BUV496 612946 HIT2 BD Biosciences
CD27 BV786 302832 O323 BioLegend
HLA-DR BV650 564231 G46-6 BD Biosciences
TIM-3 BV605 345018 F38-2E2 BioLegend
CD95 BB700 566543 DX2 BD Biosciences
CD127 BB630 Custom HIL-7R-M21 BD Biosciences
CD4 PE Cy5.5 35-0042-82 RMA-4.5 Thermo Fisher Scientific
TIGIT PE-Dazzle 372716 A15153G BioLegend
Granzyme B BB790 Custom GB11 BD Biosciences
TCF-1 AF488 6444S C63D9 Cell Signaling
T-BET PE-Cy7 25-5825-82 4B10 eBioscience
KI67 AF700 561277 B56 BD Biosciences

	EOMES EF660 50-4877-42 WD1928 eBioscience<br>Anti-mouse IgG HRP 115-005-003 polyclonal Jackson ImmunoResearch<br>Anti-SARS-CoV-2 nucleocapsid protein BSM-41411M 1C7 Stratech Scientific
Validation	All primary antibodies used in this study were commercially purchased and routinely tested by the respective manufacturers for validation in flow cytometry.

Eukaryotic cell lines

Policy information about cell lines and Sex and Gender in Research

Cell line source(s)	A549 (ATCC#CCL-185) and VeroE6 cells (ATCC#CRL-1586) expressing human angiotensin-converting enzyme 2 (ACE2) and transmembrane serine protease 2 (TMPRSS2).
Authentication	The A549 (ATCC# CCL-185) and VeroE6 (ATCC# CRL-1586) cell lines were authenticated through morphology assessment, STR (short tandem repeat) profiling. No additional authentication was performed beyond the manufacturer's validation. Cells were used at low passage numbers and routinely monitored for morphology and growth characteristics.
Mycoplasma contamination	None detected testing routinely.
Commonly misidentified lines<br>(See ICLAC register)	No commonly misidentified cell lines were used in this study.

Plants

Seed stocks	Not applicable.
Novel plant genotypes	Not applicable.
Authentication	Not applicable.

Flow Cytometry

Plots

Confirm that:

- [x] The axis labels state the marker and fluorochrome used (e.g. CD4-FITC).
- [x] The axis scales are clearly visible. Include numbers along axes only for bottom left plot of group (a 'group' is an analysis of identical markers).
- [x] All plots are contour plots with outliers or pseudocolor plots.
- [x] A numerical value for number of cells or percentage (with statistics) is provided.

Methodology

Sample preparation	PBMCs were isolated via standard density gradient centrifugation and cryopreserved in fetal bovine serum (Thermo Fisher Scientific) containing 10% dimethyl sulfoxide (Sigma-Aldrich).
Instrument	BD FACSymphony™ A3 or BD FACSymphony™ A5.
Software	BD FACSDiva version 9.1<br>FlowJo version 10.9.0
Cell population abundance	No cell sorting experiments were performed in this study.
Gating strategy	Gating strategies are shown in Extended Data Figures 2a, 2b, 4b.

- [x] Tick this box to confirm that a figure exemplifying the gating strategy is provided in the Supplementary Information.
